# Quantum transport evidence of Weyl fermions in an epitaxial ferromagnetic oxide

**DOI:** 10.1038/s41467-020-18646-8

**Published:** 2020-10-09

**Authors:** Kosuke Takiguchi, Yuki K. Wakabayashi, Hiroshi Irie, Yoshiharu Krockenberger, Takuma Otsuka, Hiroshi Sawada, Sergey A. Nikolaev, Hena Das, Masaaki Tanaka, Yoshitaka Taniyasu, Hideki Yamamoto

**Affiliations:** 1 NTT Basic Research Laboratories, NTT Corporation, Atsugi, Kanagawa 243-0198 Japan; 2 Department of Electrical Engineering and Information Systems & Center for Spintronics Research Network (CSRN), The University of Tokyo, 7-3-1 Hongo, Bunkyo-Ku, Tokyo 113-8656 Japan; 3 NTT Communication Science Laboratories, NTT Corporation, Soraku-Gun, Kyoto 619-0237 Japan; 4 Laboratory for Materials and Structures, Tokyo Institute of Technology, 4259 Nagatsuta, Midori-Ku, Yokohama, Kanagawa 226-8503 Japan; 5 Tokyo Tech World Research Hub Initiative (WRHI), Institute of Innovative Research, Tokyo Institute of Technology, 4259 Nagatsuta, Midori-Ku, Yokohama, Kanagawa 226-8503 Japan

**Keywords:** Electronic properties and materials, Magnetic properties and materials, Topological insulators

## Abstract

Magnetic Weyl semimetals have novel transport phenomena related to pairs of Weyl nodes in the band structure. Although the existence of Weyl fermions is expected in various oxides, the evidence of Weyl fermions in oxide materials remains elusive. Here we show direct quantum transport evidence of Weyl fermions in an epitaxial 4*d* ferromagnetic oxide SrRuO_3_. We employ machine-learning-assisted molecular beam epitaxy to synthesize SrRuO_3_ films whose quality is sufficiently high to probe their intrinsic transport properties. Experimental observation of the five transport signatures of Weyl fermions—the linear positive magnetoresistance, chiral-anomaly-induced negative magnetoresistance, π phase shift in a quantum oscillation, light cyclotron mass, and high quantum mobility of about 10,000 cm^2^V^−1^s^−1^—combined with first-principles electronic structure calculations establishes SrRuO_3_ as a magnetic Weyl semimetal. We also clarify the disorder dependence of the transport of the Weyl fermions, which gives a clear guideline for accessing the topologically nontrivial transport phenomena.

## Abstract (web summary)

Despite various predictions, the evidence of Weyl fermions in oxide materials remains elusive. Here, the authors show evidence of Weyl fermions in quantum transport measurements in an epitaxial ferromagnetic oxide SrRuO_3_.

## Introduction

Weyl fermions in a magnetic material have novel transport phenomena related to pairs of Weyl nodes^1–5^, and they are of both scientific and technological interest, with the potential for use in high-performance electronics, spintronics, and quantum computing. Although Weyl fermions have been predicted to exist in various oxides^6–8^, evidence for their existence in oxide materials remains elusive^9–11^. SrRuO_3_, a 4*d* ferromagnetic metal often used as an epitaxial conducting layer in oxide heterostructures^12–15^, provides a promising opportunity to seek the existence of Weyl fermions in a magnetic material. State-of-the-art oxide thin film growth technologies, augmented by machine learning techniques, may allow access to such topological matter. Here, we show direct quantum transport evidence of Weyl fermions in an epitaxial ferromagnetic oxide SrRuO_3_: unsaturated linear positive magnetoresistance (MR)^16–20^, chiral-anomaly induced negative MR^1,16,21^, π Berry phase accumulated along cyclotron orbits^16,18–20^, light cyclotron masses^16–20,22–24^ and high quantum mobility of about 10,000 cm^2^ V^−1^ s^−1^ ^16,17,22–27^. We employed machine-learning-assisted molecular beam epitaxy (MBE)^28^ to synthesize SrRuO_3_ films whose quality is sufficiently high to probe their intrinsic quantum transport properties. We also clarified the disorder dependence of the transport of the Weyl fermions, and provided a brand-new diagram for the Weyl transport, which gives a clear guideline for accessing the topologically nontrivial transport phenomena. Our results establish SrRuO_3_ as a magnetic Weyl semimetal and topological oxide electronics as a new research field.

Weyl semimetals, which host Weyl fermions described by the Weyl Hamiltonian, have intriguing and fascinating transport phenomena based on the chiral anomaly and linear band dispersion with spin-momentum locking^1–4,29^, such as chiral-anomaly induced negative MR and high mobility^16,17,21^. Compared with space-inversion-symmetry-breaking Weyl semimetals^30^, time-reversal-symmetry (TRS)-breaking ones are thought to be more suitable for spintronic applications^3,31,32^. For example, since the distribution of Weyl nodes in magnets is determined by the spin texture^1^, this distribution is expected to be controlled by the magnetization switching technique^33,34^. Recent angle-resolved photoemission spectroscopy (ARPES) studies have found experimental evidence for the electronic structure of magnetic Weyl semimetals Co_3_Sn_2_S_2_^2,3^ and Co_2_MnGa^4^, such as the presence of bulk Weyl points with linear dispersions and surface Fermi arcs. Demonstrating the relevance of Weyl fermions in a magnetic material to spintronic and electronic applications requires information on quantum oscillations, which allows us to characterize transport properties of individual orbits in a magnetic Weyl semimetal. However, systematic and comprehensive measurements of quantum transport, including the quantum oscillations, have been hampered in magnetic Weyl semimetals because of the difficulty in achieving a quantum lifetime long enough to observe them in metallic systems. Since specimens in the form of epitaxial films are advantageous for future device applications of magnetic Weyl semimetals, it is urgently required to prepare single-crystalline thin films^35^ of magnetic Weyl semimetals whose quality is sufficiently high to probe quantum transport properties.

**Fig. 1:**
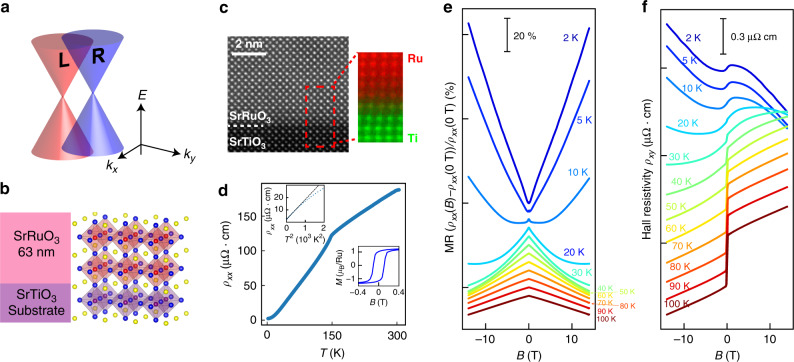
Sample characteristics and temperature dependence of the magnetoresistance and Hall resistivity of SrRuO_3_. **a** Schematic image of a pair of Weyl nodes with opposite chiralities (L and R). In ferromagnetic SrRuO_3_, the TRS breaking lifts the spin degeneracy and leads to linear band crossing at many *k* points, resulting in a pair of Weyl nodes with opposite chiralities. **b** Schematic of the sample and crystal structures of the SrRuO_3_ films (63-nm thick) on a SrTiO_3_ substrate. In the schematic crystal image, yellow, blue, red, and purple spheres indicate strontium, oxygen, ruthenium, and titanium, respectively. **c** Cross-sectional high-angle annular dark field scanning transmission electron microscopy (HAADF-STEM) image of the SrRuO_3_ film with the RRR of 71 taken along the [100] axis of the SrTiO_3_ substrate. The inset in **c** shows a color overlay of the electron energy loss spectroscopy (EELS)-STEM images for the Ti-*L*_2,3_- (green) and Ru-*M*_4,5_-edge (red). Epitaxial growth of the high-quality single-crystalline SrRuO_3_ film with an abrupt substrate/film interface is seen in the images. **d** *ρ*_*xx*_–*T* curves for the SrRuO_3_ film with the RRR of 84.3. The left inset in **d** shows the *ρ*_*xx*_ vs. *T*^2^ plot with the linear fitting (black dashed line). We defined the Fermi liquid region as the temperature range where the experimental *ρ*_*xx*_ and the fitting line are close enough to each other (<0.1 μΩ cm). The right inset in **d** shows the magnetization *M* vs. *B* curve at 10 K with *B* applied in the out-of-plane [001] direction of the SrTiO_3_ substrate. **e**, **f** MR (*ρ*_*xx*_(*B*) − *ρ*_*xx*_*(*0 T))/*ρ*_*xx*_(0 T) and Hall resistivity *ρ*_*xy*_(*B*) curves at 2–100 K for the SrRuO_3_ film with the RRR of 84.3 with *B* applied in the out-of-plane [001] direction of the SrTiO_3_ substrate. In **e** and **f**, the MR and the Hall resistivity at each temperature have been offset by 7% and 0.22 μΩ cm, respectively, for easy viewing.

Theoretically, the presence of Weyl fermions has been predicted for SrRuO_3_, a 4*d* ferromagnetic material^7^. SrRuO_3_ is widely used as an epitaxial conducting layer in oxide electronics and spintronics owing to the unique nature of ferromagnetic metal, compatibility with other perovskite-structured oxides, and chemical stability^12–15,34^. Theoretical studies predicted that the electronic structure of SrRuO_3_ may include a large number of Weyl nodes caused by the TRS breaking and spin–orbit coupling (SOC) (Fig. 1a)^7^, and suggested that the Berry phase from the Weyl nodes gives rise to an anomalous Hall effect (AHE) in it.^7,10,36^ However, a definitive conclusion on the presence of Weyl fermions near the Fermi energy (*E*_F_) cannot be drawn from observations of the AHE alone^7,9,10^, because the AHE reported so far for SrRuO_3_ can be well reproduced using a function composed of both intrinsic (Karplus–Luttinger (KL) mechanism) and extrinsic (side-jump scattering) terms^37,38^.

In this study, we conducted comprehensive high field magnetotransport measurements (see Methods section “Magnetotransport measurements”) including quantum oscillations of resistivity (i.e., Shubnikov-de Haas (SdH) oscillations), on an extraordinarily high-quality SrRuO_3_ film (63-nm thick) epitaxially grown on SrTiO_3_ (Fig. 1b, c and Supplementary Fig. 1a). Our first-principles electronic structure calculations predicted the presence of Weyl fermions within an energy range of −0.2 to 0.2 eV around the Fermi level in SrRuO_3_. To probe the contribution of the Weyl fermions to the transport properties, it is necessary to identify the following five signatures from the magnetotransport data: (1) unsaturated linear positive MR^16–20^ (2) chiral-anomaly induced negative MR^1,16,21^, (3) π Berry phase accumulated along the cyclotron orbits^16,18–20^, (4) light cyclotron mass^16,18–20,22–24^, and (5) high quantum mobility^16,17,22–27^. Although the light cyclotron mass, high mobility, and linear positive MR also exist in semiconductors with parabolic bands^39–41^, we confirmed the existence of the Weyl fermions in SrRuO_3_ by observing all of the five signatures.

## Results

### Temperature dependence of resistivity

The residual resistivity ratio (RRR), which is defined as the ratio of the longitudinal resistivity *ρ*_*xx*_ at 300 K [*ρ*_*xx*_(300 K)] and *T*$\to$0 K [*ρ*_*xx*_(*T*$\to$0 K)] (*T*: temperature), is a good measure to gauge the purity of a metallic system, that is, the quality of single-crystalline SrRuO_3_ thin films (see Methods section “Determination of the RRR in SrRuO_3_”). ﻿In fact, high RRR values are indispensable for exploring intrinsic electronic states. ﻿More specifically, RRR values above 40 and 60 have enabled observations of sharp, dispersive quasiparticle peaks near the Fermi level by ARPES^42^ and quantum oscillations via electrical resistivity^43^, respectively. To form SrRuO_3_ with quality exceeding current levels, we employed our recently developed machine-learning-assisted MBE (see Methods section “Machine-learning-assisted MBE”)^28^.

The resistivity *ρ*_*xx*_ vs. *T* curve of the SrRuO_3_ thin film shows a clear kink at the Curie temperature (*T*_C_) of ∼152 K (Fig. 1d)^12^, while the magnetization measurement at *T* = 10 K shows a typical ferromagnetic hysteresis loop (Fig. 1d, right inset). With a residual resistivity *ρ*_*xx*_ (*T*$\to$0 K) of 2.23 μΩ cm and an RRR of 84.3, SrRuO_3_ thin films grown by machine-learning-assisted MBE are superior to those prepared by any other method^12,28,43,44^. Below approximately 20 K, the *T*^2^ scattering rate ($\rho _{xx} \propto T^2$) expected in a Fermi liquid is observed (Fig. 1d, left inset)^12,42^, indicating that the intrinsic transport phenomenon is seen below this temperature (hereafter called *T*_F_).

### Temperature dependence of magnetoresistance and Hall resistivity

In this Fermi liquid region [*T* < *T*_F_ (20 K)], a semimetallic behavior is seen in the MR (*ρ*_*xx*_(*B*) − *ρ*_*xx*_(0 T))/*ρ*_*xx*_(0 T) (Fig. 1e) and Hall resistivity *ρ*_*xy*_(*B*) (Fig. 1f) curves with the magnetic field *B* applied in the out-of-plane [001] direction of the SrTiO_3_ substrate. As shown in Fig. 1e, *ρ*_*xx*_ above *T*_F_ shows the negative MR because of the suppression of magnetic scattering^12,45,46^, and the MR changes its sign below *T*_F_. Importantly, the positive MR at 2 K shows no signature of saturation even up to 14 T, which is typical of a semimetal^16,47^ and also commonly seen in Weyl semimetals^1,16–20^. Especially in the case of Weyl semimetals, linear energy dispersion of Weyl nodes is considered to be one of the most plausible origins of unsaturated linear positive MR^48,49^ (see Methods section “Excluding other possible origins of the positive MR in SrRuO_3_”). In addition, as shown in Fig. 1f, the *ρ*_*xy*_(*B*) curves below *T*_F_ are nonlinear, indicating the coexistence of multiple types of carriers (electrons and holes). We note that, below *T*_F_, the AHE, which stems from the extrinsic side-jump scattering and intrinsic KL mechanisms in SrRuO_3_,^37,38^ is well suppressed due to the small residual resistivity of the SrRuO_3_ film with the RRR of 84.3, and thus the *ρ*_*xy*_(*B*) curves below *T*_F_ are dominated by the ordinary Hall effect (see Methods section “Temperature dependence of the AHE in the SrRuO_3_ film with the RRR = 84.3”). Below 10 K, where the AHE is negligible, both the *ρ*_*xy*_(*B*) values and the slopes of *ρ*_*xy*_(*B*) change their signs in the high-*B* region, signaling the possibility of the coexistence of high-mobility electrons with low-mobility holes^16^. Importantly, both the unsaturated linear positive MR and nonlinear Hall resistivity features start to appear simultaneously when the measurement temperature is decreased to the Fermi liquid range [*T* < *T*_F_ (20 K)]. This indicates that the unsaturated linear positive MR stems from the electron- and hole-like Weyl fermions.

### Angle dependence of magnetoresistance

**Fig. 2:**
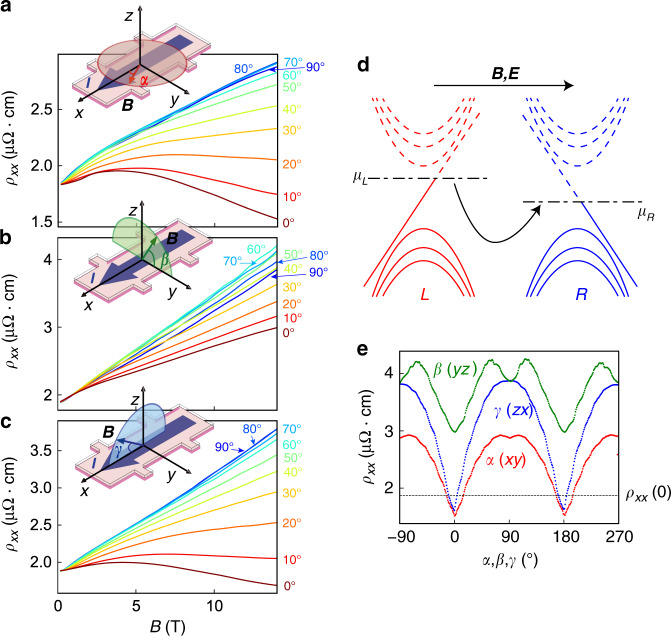
Chiral anomaly in the transport of SrRuO_3_. **a**–**c**, MR *ρ*_*xx*_(*B*) at *B* angles *α* (**a**), *β* (**b**), and *γ* (**c**) at 2 K for the SrRuO_3_ film with the RRR of 84.3. Angles *α*, *β*, and *γ* are defined in the insets of (**a**–**c**). **d** Schematic image of the chiral-anomaly induced negative MR. ﻿Red and blue bands represent Landau levels of a pair of Weyl nodes with opposite chiralities L and R, respectively. Non-orthogonal electric and magnetic fields (***E***·***B*** ≠ 0) lead to the chiral charge transfer between the two Weyl nodes with opposite chiralities (L and R). The currents of the chiral charges are observed as a form of negative MR. Here, *μ*_L_ and *μ*_R_ indicate chemical potentials in the Weyl points with opposite chiralities. **e** *α-*, *β-*, and *γ-*angular dependences of the MR *ρ*_*xx*_ with *B* = 14 T at 2 K for the SrRuO_3_ film with the RRR of 84.3. The black dashed line indicates the *ρ*_*xx*_ (0 T) value. The region below the black dashed line shows negative MR.

Next, we observed the chiral-anomaly induced negative MR, which is an important signature of Weyl fermions^1,16,21,50^. To clarify the anisotropic character of the chiral-anomaly induced negative MR thoroughly and systematically, we measured *ρ*_*xx*_(*B*) at *B* angles *α*, *β*, and *γ* in the *xy*, *yz*, and *zx* planes, respectively (Fig. 2a–c). The rotation angles *α*, *β*, and *γ* are defined in the insets of Fig. 2a–c. When *B* is applied perpendicular to the current *I* (*B*⊥*I*, *α* = 90° or *β* = 0-90° or *γ* = 90°), the unsaturated linear positive MR is observed. The unsaturated linear positive MR seen here is expected owing to the presence of the Weyl fermions, and those states are supposedly anisotropic because of the $\sim$0.5% compressive strain of SrRuO_3_ induced by the SrTiO_3_ substrates^28^. This anisotropy is confirmed by varying *β* (Fig. 2b). In contrast, when *B* is rotated parallel to the current (*B//I*, *α* = 0° or *γ* = 0°), the MR turns negative and becomes linear above 8 T (Fig. 2a, c and Supplementary Fig. 4c). Theoretical calculations based on the semiclassical Boltzmann kinetic equation predict that TRS-breaking Weyl semimetals show a negative MR that is linear in *B*^1,50^, in comparison with the quadratic dependence expected for space-inversion-symmetry-breaking Weyl semimetals^16,21^. Thus, the observed linear increase of the negative MR is consistent with a chiral anomaly in magnetic Weyl semimetals. The chiral-anomaly induced negative MR can be understood from the violation of the conservation rules of chiral charges as shown in Fig. 2d^50^, and it should be maximum when *B* is exactly parallel to *I* (*α* = 0°, 180°, or *γ* = 0°, 180°). As expected, this anisotropic feature of the negative MR is observed at *α* = 0°, 180° or *γ* = 0°, 180° under 14 T (Fig. 2e). In addition, the peak structures in the angle dependence of the negative MR at *B*//*I* (*α* = 0°, 180° or *γ* = 0°, 180°) are similar to those in previous observations of the chiral anomaly in other Weyl semimetals^1,16,21,51,52^. These results confirm that this linear negative MR is induced by the chiral anomaly (see Methods section “Excluding other possible origins of the negative MR in SrRuO_3_”). As a consequence of the contributions from the positive MR and negative MR, *ρ*_*xx*_(*B*) is lower than the zero field resistivity *ρ*_0_ = *ρ*_*xx*_(0 T) when *α* and *γ* are near 0° or 180°.

### Quantum oscillations

**Fig. 3:**
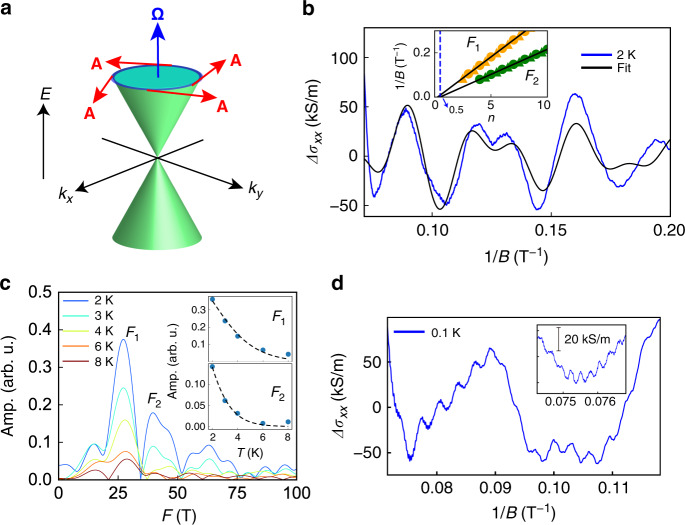
Quantum oscillations of SrRuO_3_. **a** Schematic image of energy dispersion near a Weyl point. Blue and red arrows represent the Berry curvature *Ω* and the Berry connection *A*, respectively. The π Berry phase is accumulated along the cyclotron orbits (blue circle). **b** SdH oscillation measured at 2 K with *B* (5 T < *B* < 14 T) applied in the out-of-plane [001] direction of the SrTiO_3_ substrate for the SrRuO_3_ film with the RRR of 84.3. Black curve is the fitting curve of Eq. (1). The fitting was carried out by a nonlinear least squares method with the fitting parameters *A*_1_, *β*_1_, *T*_*D*1_, *A*_2_, *β*_2_, and *T*_*D*2_. The inset shows fan diagrams of two oscillation components of *F*_1_ (=26 T) and *F*_2_ (=44 T), which are shown as orange and green symbols, respectively. Here, the circles and triangles indicate integer and half-integer indexes of the oscillation components. **c** Fourier transform spectra of the SdH oscillations at 2–8 K. Insets in **c** show the mass estimations of the *F*_1_ and *F*_2_ orbits according to the LK theory. Black dashed curves are the fitting curves. **d** SdH oscillation observed at 0.1 K for the SrRuO_3_ film with the RRR of 84.3. The inset in **d** shows a close-up at around 0.075 T^−1^. The oscillation holds four kinds of other trivial orbits with higher frequencies.

The Berry phase has become an important concept in condensed matter physics over the past three decades, since it represents a topological classification of the system and it plays a fundamental role in various phenomena, such as electric polarization, orbital magnetism, etc.^53^. However, revealing the π Berry phase, originating from a band touching point of the Weyl node^16^, has been challenging in magnetic Weyl semimetals. Here, we detect the π Berry phase accumulation along the cyclotron orbits, for the first time in magnetic Weyl semimetals, by measurement techniques sensitive to the quantized energy levels, i.e., SdH oscillation. The nontrivial π Berry phase, which is acquired by a surface integral of the Berry curvature Ω over a closed surface containing a Weyl point in the *k*-space (Fig. 3a)^16,18–20^, causes a phase shift of quantum oscillations. According to the Lifshitz–Kosevich (LK) theory, the magnitude of the SdH oscillation is described as^16,18–20,54^

1 $$\begin{array}{*{20}{c}} {{\Delta}\sigma _{xx} = \mathop {\sum }\limits_i A_i\frac{{X_i}}{{\sinh X_i}}\exp \left( { - \frac{{2\pi ^2k_BT_{Di}}}{{\hbar \omega _{ci}}}} \right)\cos \left[ {2\pi \left( {\frac{{F_i}}{B} - \frac{1}{2} + \beta _{{\mathrm{B}}i}} \right)} \right]} \end{array},$$

where *Δσ*_*xx*_ is the oscillation component of the longitudinal conductivity, *A*_*i*_ is the normalization factor, *X*_*i*_ = 2*π*^2^*k*_*B*_*T*/*ħω*_*ci*_, *k*_*B*_ is the Boltzmann constant, *ħ* is the reduced Planck constant, *ω*_*ci*_ is the cyclotron frequency defined as *eB*/*m*_i_^*^, *m*_i_^*^ is the cyclotron mass, *T*_*Di*_ is the Dingle temperature, *F*_*i*_ is the frequency of the SdH oscillation, and 2π*β*_*Bi*_ is the phase shift caused by the Berry phase as mentioned above. The subscript *i* is the label of an orbit of carriers. Note that, even when a large number of carriers in 3D multiband systems pin the chemical potential, the linear dispersion of the Weyl fermions leads to an unconventional $\pi$ phase shift in the SdH oscillation (see Methods section “Effect of the Fermi-level pinning on the phase shift in the SdH oscillation”). To extract the Berry phases in SrRuO_3_, we used the LK formula [Eq. (1)] for two frequencies to fit the SdH oscillation data. Figure 3b shows the SdH oscillation data at 2 K and the fitting results by Eq. (1). (see Methods section “Data pretreatment for quantum oscillations”). The oscillation spectrum is considerably complex because of the contribution from several subbands^7^. To reduce the fitting parameters, we first carried out fast Fourier transform of the SdH oscillation (Fig. 3c), and extracted the *F*_1_ (26 T), *F*_2_ (44 T), *m*_1_^*^ (0.35*m*_0_), and *m*_2_^*^ (0.58*m*_0_) (*m*_0_, electron rest mass) values from the peak positions and the temperature dependence of *F*_1_ and *F*_2_ based on the LK theory (Fig. 3c, insets). Small masses are expected for Weyl fermions, which would fulfill the light cyclotron mass signature^16,18–20,22–24^. From the fitting to the data at 0.07 T^−1^ < 1/*B* < 0.2 T^−1^ shown in Fig. 3b, we obtained *T*_*D*1_ = 0.63 K, *T*_*D*2_ = 0.34 K, *β*_B1_ = 0.27, and *β*_B2_ = 0.48. The 2*πβ*_B2_ value of 0.96*π* indicates the presence of the nontrivial π Berry phase arising from the mass-less dispersion of the Weyl fermions (see Methods section “Exclusion of other possible mechanisms of the phase shift in SrRuO_3_”). Although the interpretations of phase shifts between 0 and π in topological materials are still controversial, the 2*πβ*_B1_ value of 0.54*π* implies that the energy dispersion of the *F*_1_ orbit has both quadratic (trivial) and linear (nontrivial) features^55,56^. These results cannot be reproduced by fixed zero Berry phases, confirming the existence of a nonzero Berry phase (Supplementary Fig. 6a).

The SdH oscillations not only give an insight into the topological nature, but also provide evidence of very high mobility of the Weyl fermions enclosing the Weyl points. We quantitatively determine the mobility of the charge carriers of the *F*_1_ and *F*_2_ orbits by calculating the quantum mobility, *µ*_*qi*_ = e*ħ*/(2*πk*_*B*_*m*_i_^*^T_*Di*_). The obtained *µ*_*q*1_ and *µ*_*q*2_ values are 9.6 × 10^3^ and 1.1 × 10^4^ cm^2^ V^−1^ s^−1^, respectively. In addition, assuming isotropic Weyl nodes, we can estimate the carrier concentrations *n*_*i*_ = $\frac{1}{{6\pi ^2}}\left( {\frac{{2eF_i}}{\hbar }} \right)^{\frac{3}{2}}$ (*n*_1_ = 3.8$\times$10^17^ ${\mathrm{cm}}^{ - 3}$ and *n*_2_ = 8.3$\times$10^17^ ${\mathrm{cm}}^{ - 3}$) and the chemical potentials *µ*_*i*_ = *eħF*_*i*_/*m*_i_^*^ (*µ*_1_ = 8.5 meV and *µ*_2_ = 8.8 meV).^57^ These results mean that *F*_1_ and *F*_2_ come from the high-mobility and low-concentration Weyl fermions enclosing the Weyl points.

In addition to the Weyl fermions as evidenced by the five signatures described above, there are trivial Ru 4*d* bands crossing the *E*_F_ in SrRuO_3_^7,12,42,43^. SdH experiments performed at 0.1 K (Fig. 3d) confirmed some of these trivial Fermi pockets with *F*_3_ = 300 T, *F*_4_ = 500 T, *F*_5_ = 3500 T, and *F*_6_ = 3850 T (see Methods section “SdH oscillations of trivial orbits”). These four orbits have heavier masses (>2.8*m*_0_) than those of *F*_1_ and *F*_2_, which are consistent with the reported values for SrRuO_3_^12,43^. The Fermi pocket areas of *F*_3_ (0.029 Å^−2^) and *F*_4_ (0.048 Å^−2^) are close to those of the 364 T (0.035 Å^−2^) orbit reported in earlier de Haas–van Alphen measurements^58^, and the Fermi pocket areas of *F*_5_ (0.334 Å^−2^), and *F*_6_ (0.368 Å^−2^) correspond to the $\alpha$_1_ band (0.33–0.37 Å^−2^) observed by the ARPES and in earlier SdH measurements^42,43^. Noteworthy is that the Fermi pocket areas of *F*_1_ (0.0025 Å^−2^) and *F*_2_ (0.0042 Å^−2^) are more than ten times smaller than those of the trivial orbits, indicating that *F*_1_ and *F*_2_ stem from the small Fermi pockets as a feature of the Weyl fermions.

### Disorder dependence of transport properties

**Fig. 4:**
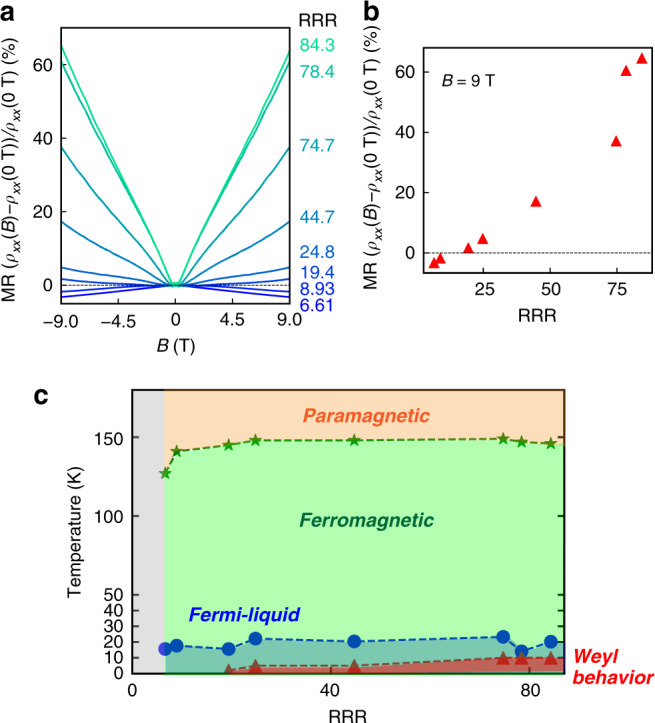
RRR dependence of the transport phenomena in SrRuO_3_. **a** RRR dependence of the MR (*ρ*_*xx*_(*B*) − *ρ*_*xx*_(0 T))/*ρ*_*xx*_(0 T) measured at 2 or 2.3 K. When RRR > 19.4, the positive MR, which is one piece of experimental evidence of the Weyl fermions in SrRuO_3_, clearly emerges. **b** RRR dependence of the MR (*ρ*_*xx*_(*B*) − *ρ*_*xx*_ (0 T))/*ρ*_*xx*_ (0 T) at *B* = 9 T and *T* = 2 or 2.3 K. **c** Diagram of the RRR dependence of *T*_C_, *T*_F_, and *T*_W_, which are shown as green stars, blue circles, and red triangles, respectively.

Observing the intrinsic transport signatures of the Weyl fermions requires a high-quality SrRuO_3_ sample, since it is easily hindered by disorders such as defects and impurities. To show the disorder dependence of the transport phenomena in SrRuO_3_, we investigated the RRR dependence of *T*_C_, *T*_F_, and the highest temperature where the linear positive MR, one of the clear features of Weyl fermion transport in SrRuO_3_, remains (hereafter called *T*_W_) (Fig. 4a–c) (see Methods section “RRR dependence of the ferromagnetism, Fermi liquid behavior, and Weyl behavior”). As shown in Fig. 4c, the ferromagnetism becomes weaker (*T*_C_ < 150 K) below RRR = 8.93, the Fermi liquid behavior remains regardless of the RRR values even with low RRR = 6.61, and the positive MR is observed over the RRR = 19.4. It is remarkable that the positive MR ratio at 9 T increases with increasing RRR (Fig. 4a, b), which indicates that the Weyl fermions become more dominant in the transport. In the RRR dependence of the Hall resistivity, *ρ*_*xy*_(*B*), the nonlinear *B* dependence becomes more prominent with a sign change in *dρ*_*xy*_/*dB* with increasing RRR (see details in Methods section “RRR dependence of the Hall resistivity”). Thus, a high-quality SrRuO_3_ sample is essential for observing the intrinsic transport of the Weyl fermions, and the diagram presented in Fig. 4c will be an effective guideline for realizing topologically nontrivial transport phenomena of the Weyl fermions to connect magnetic Weyl semimetals to spintronic devices^31,32^.

### Electronic structure calculations

**Fig. 5:**
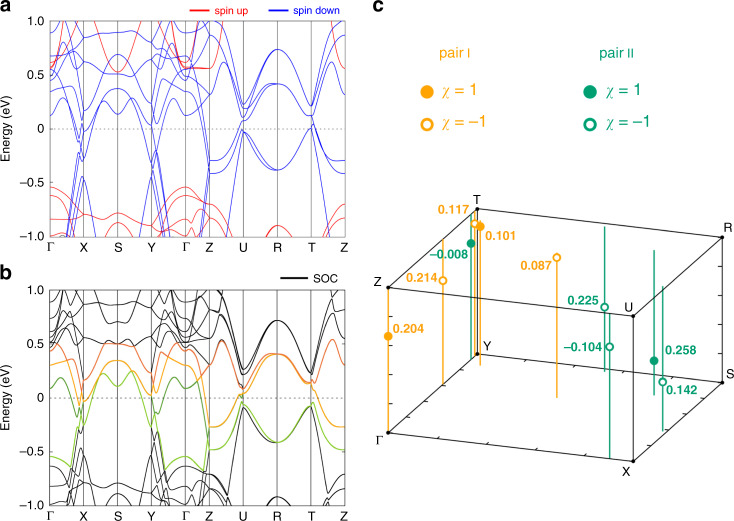
Predicted Weyl points in the orthorhombic *Pbnm* (*D*_*2h*_) phase of SrRuO_3_. **a** Band structure for the ferromagnetic ground state without spin-orbit coupling as obtained from GGA + *U* calculations with *U* = 2.6 eV and *J* = 0.6 eV. **b** Band structure for the ferromagnetic state with spin–orbit coupling and the magnetization along the orthorhombic *c*-axis obtained from GGA + *U* + SOC calculations with *U* = 2.6 eV and *J* = 0.6 eV. Two pairs of bands I (orange and dark orange bands) and II (green and dark green bands) are selected for calculating the corresponding chirality $\chi$ at each band crossing point. **c** Positions of the Weyl points in the irreducible part of the Brillouin zone calculated for two pairs of bands I (orange filled and open circles for $\chi$ = 1 and −1, respectively) and II (green filled and open circles for $\chi$ = 1 and −1, respectively). Numbers next to the points indicate their energy distance from the Fermi level (*E* − *E*_F_, where *E*_F_ is the Fermi energy) in eV unit. Vertical orange and green lines indicate the in-plane (Γ–X–S–Y plane) positions of the Weyl points for easy viewing. Note that the orange and dark green bands are degenerate at the border of the Brillouin zone (X–S–Y and Z–U–R–T–Z).

Thus far, the magnetotransport data of a high-quality SrRuO_3_ shows all of the expected marks of a magnetic Weyl semimetal. For certainty and theoretical rigor, we performed first-principles electronic structure calculations (see Methods section “Computational details”) to analyze the energy dispersion of SrRuO_3_. The calculated electronic structure for the orthorhombic *Pbnm* phase of SrRuO_3_ for the ferromagnetic ground state shows a half-metallic behavior (Fig. 5a) which agrees with previous electronic structure calculations^59^. The bands near the Fermi level are formed by the *t*_*2g*_ Ru states hybridized with the O 2*p* orbitals (Supplementary Fig. 8a). The calculated magnetic moment per Ru ion (~1.4 $\mu _B$, as tabulated in Supplementary Table 2) is close to the experimental saturation magnetic moment of 1.25 $\mu _B$/Ru of our SrRuO_3_ films.^28^ We observe that in the ferromagnetic phase the Ru spins tend to align along the crystallographic *b* axis reducing the symmetry of the system from *D*_*2h*_ to *C*_*2h*_. To identify the existence of the Weyl points (the band crossing points that carry $\pm 1$ chiral charge) by evaluating the outward Berry flux enclosed in a small sphere, we examined each band crossing of two pairs of bands I and II, shown in Fig. 5b, near the Fermi level in the presence of SOC with the magnetization along the orthorhombic *c*-axis. The resulting Weyl points are shown and listed in Fig. 5c and Supplementary Tables 3 and 4, respectively. We identified a total of 29 pairs of Weyl points with opposite chirality in the full Brillouin zone (BZ). An earlier theoretical study also made similar predictions of the existence of Weyl points in the case of a cubic structure of SrRuO_3_.^7^ Most of these Weyl points are found to exist within an energy range of −0.2 to 0.2 eV around the Fermi level. Among them, $\left| {E - E_F} \right|$ for WP_z_6_1–4_ (8–16 meV) in Supplementary Table 4 is very close to the experimental chemical potentials of the Weyl fermions estimated from the SdH oscillations (*µ*_1_ = 8.5 meV and *µ*_2_ = 8.8 meV for the *F*_1_ and *F*_2_ orbitals, respectively). The Weyl points near the Fermi level are expected to contribute to all the observed quantum transport phenomena reported in this study. It is important to note that a small monoclinic distortion induced by the substrate does not significantly change the band structure of SrRuO_3_ and that Weyl points are robust with the distortion as long as the inversion symmetry is present (see Method section “First-principles calculations of Weyl points”), leading to a congruence of the experimental findings with theoretical predictions.

In conclusion, we have observed the emergence of Weyl fermions in epitaxial SrRuO_3_ film with the best crystal quality ever reported^12^, which was grown by machine-learning-assisted MBE. Experimental observation of the five important transport signatures of Weyl fermions—the linear positive MR, chiral-anomaly induced negative MR, *π* phase shift in a quantum oscillation, light cyclotron mass, and high quantum mobility of about 10,000 cm^2^ V^−1^ s^−1^—combined with first-principles electronic structure calculations establishes SrRuO_3_ as a magnetic Weyl semimetal. In addition, the RRR dependences of ferromagnetism, Fermi liquid behavior, and positive MR serve as a road map to merge two emerging fields: topology in condensed matter and oxide electronics. Our results will pave the way for topological oxide electronics.

## Methods

### Magnetotransport measurements

We first deposited the Ag electrodes on a SrRuO_3_ surface. Then, we patterned the samples into 200 × 350 μm^2^ Hall bar structures by photolithography and Ar ion milling. Resistivity was measured using the four-probe method at 100 μA in a Physical Property Measurement System (PPMS) DynaCool sample chamber equipped with a rotating sample stage. Low-noise measurements below 1 K were performed by an AC analog lock-in technique, and the sample was cooled down in a ^3^He–^4^He dilution refrigerator.

### Determination of the RRR in SrRuO_3_

The RRR was determined as the ratio of the longitudinal resistivity *ρ*_*xx*_ at 300 K [*ρ*_*xx*_(300 K)] and *T*$\to$0 [*ρ*_*xx*_(*T*$\to$0 K)]. SrRuO_3_ exhibits Fermi liquid behavior at a low temperature, which is characterized by a linear relationship between *ρ*_*xx*_ and *T*^2^.^12,42^ Based on this relationship, we estimated *ρ*_*xx*_ (*T*$\to$0 K) by extrapolating the *ρ*_*xx*_ vs. *T*^2^ fitting line below 10 K (Supplementary Fig. 1b).

### Machine-learning-assisted MBE

We grew the high-quality SrRuO_3_ films (63-nm thick) on (001) SrTiO_3_ substrates in a custom-designed MBE setup equipped with multiple e-beam evaporators for Sr and Ru (Supplementary Fig. 2a). We precisely controlled the elemental fluxes, even those of elements with high melting points, e.g., Ru (2250 °C), by monitoring the flux rates with an electron-impact-emission-spectroscopy sensor and feeding the results back to the power supplies for the e-beam evaporators. The oxidation during growth was carried out with ozone (O_3_) gas. O_3_ gas ($\sim$15% O_3_ + 85% O_2_) was introduced through an alumina nozzle pointed at the substrate. Further information about the MBE setup and preparation of the substrates is described elsewhere^60^. The surface morphology of our SrRuO_3_ films is composed of atomically flat terraces and steps, as observed by atomic force microscopy^28^. Together with Laue fringes in the *θ*–2*θ* X-ray diffraction patterns^28^, this indicates the high crystalline order and large coherent volume of our SrRuO_3_ films.

Fine-tuning of growth conditions is essential but challenging for high-RRR SrRuO_3_ growth. Therefore, only a few papers have reported SrRuO_3_ films with RRRs over 50.^28,43,44^ While a conventional trial-and-error approach may be a way to optimize the growth conditions, this is time-consuming as well as costly, and the optimization efficiency largely depends on the skill and experience of individual researchers. To avoid such a time-consuming approach and reduce experimental time and cost, we employed machine-learning-assisted MBE, which we developed in previous research^28^. Here, three important growth parameters [Ru flux rate, growth temperature, and nozzle-to-substrate distance (Supplementary Fig. 2a)] were optimized by a Bayesian optimization (BO) algorithm, which is a sample-efficient approach for global optimization^61^. This algorithm sequentially produces the three parameter values at which a high RRR value is predicted given past trials.

Supplementary Figure 2b shows the procedure for machine-learning-assisted MBE growth of the SrRuO_3_ thin films based on the BO algorithm. Here, we optimized each parameter in turn using BO. We first chose one of the growth parameters to update and fixed the rest, ran the BO algorithm to search the growth parameter, and then switched to another growth parameter. This is because BO can be inefficient in large dimensions due to the difficulty of predicting the outcome value for unseen parameters. Here, RRR = *S*(*x*) is the target function specific to our SrRuO_3_ films, and *x* is the growth parameter (Ru flux rate, growth temperature, or nozzle-to-substrate distance). BO constructs a model to predict the value of *S*(*x*) for unseen *x* using the result of past *M* trials $\left\{ {x_m,\,{\mathrm{RRR}}_m} \right\}_{m \,=\, 1}^M$, where *x*_*m*_ is the *m*th growth parameter and RRR_*m*_ is the corresponding RRR value. Specifically, we use the Gaussian process regression (GPR) as a prediction model^28,62^. GPR predicts *S*(*x*) as a Gaussian random variable following *N* ($\mu$, $\sigma ^2$). This means $\mu$ and $\sigma ^2$ are calculated from *x* and the *M* data points. Subsequently, we choose the growth parameter *x* in the next run such that the expected improvement (EI)^63^ is maximized. EI balances the exploitation and exploration by using the predicted $\mu$ and $\sigma ^2$ at *x*. This measures the expectation of improvement over the best RRR observed so far. This routine is iterated until further improvement is no longer expected. In practice, we terminate the iteration when the number of trials reaches the predetermined budget. Here, we stopped the routine at 11 samplings per parameter. After completing 11 samplings for a certain parameter, we chose the value that gave the highest RRR and started the optimization of another parameter. In this optimization procedure, we used RRR (*T* = 4 K) instead of RRR (*T*$\to$0 K) for easy estimation. Further details about the implementation of machine-learning-assisted MBE are described elsewhere^28^.

In our previous study^28^, we carried out the optimization of the Ru flux rate while keeping the other parameters unaltered. Subsequently, we tuned the growth temperature and the nozzle-to-substrate distance. As a result, we obtained a high-RRR (51.8) SrRuO_3_ film in only 24 MBE growth runs (Supplementary Fig 2c). Since the RRR was still lower than the highest value reported ($\sim$80) in the literature^12,44^, we further carried out the re-optimization of the Ru flux rate and growth temperature with previously optimized parameters (the Ru flux = 0.42 Å s^−1^, growth temperature = 721 °C, and nozzle-to-substrate distance = 15 mm) as a starting point to find the global-best point in the three-dimensional parameter space. Supplementary Figure 2c shows the highest experimental RRR values plotted as a function of the total number of MBE growth runs. With the re-optimization of the Ru flux rate and growth temperature, the highest RRR (*T* *=* 4 K) value increased and reached 81 in only 44 MBE growth runs. The highest experimental RRR (*T*$\to$0 K), 84.3, was achieved at the Ru flux = 0.365 Å s^−1^, growth temperature = 781 °C, and nozzle-to-substrate distance = 15 mm. The availability of such high-quality film allowed us to probe the intrinsic transport properties of SrRuO_3_.

### Excluding other possible origins of the positive MR in SrRuO_3_

Since SrRuO_3_ has complex Fermi surfaces with both topologically trivial and nontrivial bands, the contribution from the trivial bands to the MR, such as orbital MR (∝*B*^2^)^64^, anisotropic MR (AMR) (∝ relative angle between the electric current and the magnetization), and weak anti-localization (WAL) (∝*B*^−1/2^)^65^, will appear in the MR in our SrRuO_3_ films. In fact, the AMR feature is clearly observed in the near-zero field region for the SrRuO_3_ film with the RRR of 84.3 as shown in Supplementary Fig. 3a. While it is difficult to distinguish each contribution on the MR in a low-field region (<0.5 T), the MR in a high field region (>0.5 T) is clearly dominated by the unsaturated linear positive MR. The MR curve at 2 K for the SrRuO_3_ film with the RRR of 84.3 with *B* applied in the out-of-plane [001] direction of the SrTiO_3_ substrate (Supplementary Fig. 3b) is linear above 0.5 T. This means that the magnetic field dependences of the WAL (∝*B*^−1/2^) and of the orbital MR (∝*B*^2^) provide only a very limited contribution to the net MR response and that the WAL and the orbital MR are negligibly small above 0.5 T. In addition, the contribution of the AMR above 0.5 T is also negligible since the magnetization of the SrRuO_3_ films saturates at about 0.5 T (see Supplementary Fig. 3c).

Next, the linear positive MR caused by carrier fluctuations^39,48,66–70^, originating from disorders and/or non-uniformity of dopants, can be excluded in the SrRuO_3_ film with the RRR of 84.3, since its Hall resistivity is not a linear function of *B* as shown in Supplementary Fig. 3d. The carrier fluctuations can cause the linear positive MR by an admixture of a component of the Hall resistivity to the longitudinal resistivity since the mobility fluctuation makes the Hall voltage contribute to the longitudinal voltage. In ref. ^39^, the admixture of a component of the Hall resistivity to the longitudinal resistivity *ρ*_*xx*_ due to the carrier fluctuations is expressed as

2 $$\begin{array}{*{20}{c}} {\rho _{xx} \propto \frac{{d\rho _{xy}}}{{dB}} \times |B|} \end{array},$$

where *ρ*_*xy*_ is the Hall resistivity, and *B* is the external magnetic field. Therefore, *ρ*_*xx*_ is proportional to *B* when *dρ*_*xy*_/*dB* is constant. For example, the GaAs 2DEG system shows clear linear MR reflecting its high-linearity of *ρ*_*xy*_ in the whole measurement range (0–33 T)^39^. In our case, however, the Hall resistivity shows nonlinear behavior reflecting its semi-metallic feature (Supplementary Fig. 3d). In fact, the $d\rho _{xy}/dB \times |B|$ curve calculated by the *ρ*_*xy*_ curve (Supplementary Fig. 3e) does not agree with the measured MR (*ρ*_*xx*_(*B*)−*ρ*_*xx*_(0 T))/*ρ*_*xx*_(0 T) in the SrRuO_3_ film, especially in the low-magnetic-field range (0 T < *B* < 4 T), where the nonlinearity of the Hall resistivity (Supplementary Fig. 3d) is prominent. This means that carrier fluctuations are not the origin of the linear positive MR in our SrRuO_3_ films.

The admixture of a component of the Hall resistivity to the longitudinal resistivity is also ruled out by taking the small Hall resistivity of SrRuO_3_ into consideration. For the admixture of the Hall voltage, to cause the large positive MR over 100%, a large Hall voltage due to a small carrier density is necessary; only semiconducting materials can show this type of large MR. For example, the GaAs 2DEG system with the small sheet carrier density of 3 × 10^11^ cm^−2^ shows by far larger linear MR (~10^5^%)^39^ than that of Bi_2_Se_3_ (~1%) with the large sheet carrier density of 1.7 × 10^16^ cm^−2^.^68^ In contrast, since the SrRuO_3_ film with the RRR of 84.3 is metallic, the Hall resistivity (=0.36 μΩ cm) at 2 K and 14 T is more than ten times smaller than the longitudinal resistivity (=3.79 μΩ cm) at 2 K and 14 T. This means that the longitudinal voltage in SrRuO_3_ is less affected by the Hall voltage.

Therefore, we concluded that the observed unsaturated linear positive MR above 0.5 T originates from the linear energy dispersion around the Weyl nodes and that the contribution from the conventional bands on the MR is negligible in the SrRuO_3_ film with the RRR of 84.3.

### Temperature dependence of the AHE in the SrRuO_3_ film with the RRR = 84.3

It is known that the AHE in SrRuO_3_ is caused by an extrinsic factor (side-jump scattering) in addition to an intrinsic factor (KL mechanism)^12,37,38^. To determine the contributions of the intrinsic and extrinsic factors to the AHE, we investigated the temperature-dependent scaling of the AHE in the SrRuO_3_ film with the RRR = 84.3.

The Hall resistivity *ρ*_*xy*_(*B*) in SrRuO_3_ is described as the summation of the ordinary $\rho _{xy}^{{\mathrm{OHE}}}\left( B \right)$ and anomalous $\rho _{xy}^{{\mathrm{AHE}}}\left( B \right)$ components of Hall effects^12,37,38^

3 $$\begin{array}{*{20}{c}} {\rho _{xy}\left( B \right) = \rho _{xy}^{{\mathrm{OHE}}}\left( B \right) + \rho _{xy}^{{\mathrm{AHE}}}\left( B \right)} \end{array}.$$

$\rho _{xy}^{{\mathrm{AHE}}}\left( B \right)$ is proportional to the perpendicular magnetization component *M*_⊥_: $\rho _{xy}^{{\mathrm{AHE}}}\left( B \right)$ = *cρ*_*s*_*M*_⊥_(*c*; constant). Here, the proportional coefficient *ρ*_*s*_ differs depending on the origin of the AHE, which can be of intrinsic (KL mechanism) and extrinsic (side jump scattering) origin^12,37,38^. As shown in Supplementary Fig. 4a, above *T*_F_ (~20 K), clear AHE, which is proportional to the magnetization hysteresis curve (e.g., Fig. 1d, right inset), is discernible, and it dominates Hall resistivity in a near-zero magnetic field. On the other hand, below *T*_F_, the AHE components are negligibly small.

The temperature dependence of the AHE in SrRuO_3_ has been well reproduced by a model where both the intrinsic KL mechanism and the extrinsic side-jump scattering terms are taken into account^38^. In this model, the relationship between *ρ*_*s*_ and *ρ*_*xx*_ is described as

4 $$\begin{array}{*{20}{c}} {\rho _s = \frac{{a_1}}{{{\mathrm{{\Delta}}}^2 + a_2\rho _{xx}^2}}\rho _{xx}^2 + a_3\rho _{xx}^2} \end{array},$$

where *a*_1_–*a*_3_, and Δ are the fitting parameters, which are associated with the band structure^38^. The first term describes a contribution from the off-diagonal matrix elements of the velocity operators, called the KL term in the model^38^, and the second term is the contribution from the side-jump scattering. In this model, the KL term is obtained by incorporating the finite scattering rate, which is inversely proportional to $\rho _{xx}$, into Kubo’s formula^71^, and the constant *a*_1_ in Eq. (4) is expressed as

5 $$\begin{array}{*{20}{c}} {a_1 = bIm\left( A \right),} \end{array}$$

where *b* is a constant and $A$ is expressed as

6 $${A = \mathop {\smallint }\limits_{\Bbb K} \frac{{d^3k}}{{2\pi ^3}}\langle1k{\mathrm{|}}v_y{\mathrm{|}}2k\rangle\langle2k{\mathrm{|}}v_x{\mathrm{|}}1k\rangle.}$$

Here, $A$ can be associated with a Berry phase^72^. The *v*_*y*_ and *v*_*x*_ are the velocity operators in the direction of *y* and *x* axes, respectively, ${\Bbb K}$ is a set of quasi-momentum of the states producing the dominant contribution to $A$, and $k \in {\Bbb K}$. Here, “1” and “2” are indices to denote two individual bands: the former crosses the Fermi level, while the latter is fully occupied. When the system has multiple bands crossing the *E*_F_, the $a_1$ value is expressed by the sum of the $A$ values for each band. Therefore, the relationship between $\rho _s$ and $\rho _{xx}$ (Eq. (4)) does not even change in the system with multiple bands. Accordingly, this model can be applied to analyze the temperature dependence of the AHE of SrRuO_3_, although detailed information about the electronic bands near *E*_F_ in SrRuO_3_ is required to assign the contribution of each band to the intrinsic AHE.

Supplementary Figure 4b shows the *ρ*_*s*_ vs. *ρ*_*xx*_ plot of the SrRuO_3_ film with the RRR = 84.3 and the fitting result of Eq. (4). The fitting curve reproduces the AHE sufficiently, indicating that the AHE in the SrRuO_3_ film arises from the intrinsic KL mechanisms along with the extrinsic side-jump scattering. The important point in Eq. (4) is that the AHE is asymptotic to zero when *ρ*_*xx*_$\to$0. Accordingly, for the SrRuO_3_ film with such a very high RRR (84.3), equivalently with a very small residual *ρ*_*xx*_, AHE becomes negligibly small at low temperatures. Therefore, *ρ*_*xy*_(*B*) curves below *T*_F_ in our data are dominated by the ordinary Hall effect.

### Excluding other possible origins of the negative MR in SrRuO_3_

Here, we exclude other possible mechanisms as the origin of the anisotropic linear negative MR at 2 K for the SrRuO_3_ film with the RRR of 84.3. As described in the Methods section “Excluding other possible origins of the positive MR in SrRuO_3_”, the contribution from the trivial bands to the MR, such as orbital MR^64^, AMR, and WAL^65^, are negligible above 0.5 T.

The electron-magnon scattering^73^ is also excluded as an origin of the linear negative MR (Fig. 2). The measurement temperature (2 K) of the anisotropic linear negative MR is too low to excite magnons in SrRuO_3_. A recent inelastic neutron scattering study on single-crystalline SrRuO_3_^74^ reveals that the magnon gap of SrRuO_3_ is equal to 11.5 K. Therefore, the contribution from magnon scattering to the negative MR is negligible.

Finally, the boundary scattering effect is also excluded. The values of mean free paths *l*_*m*_ for the Weyl fermions (*F*_1_ (=26 T) and *F*_2_ (=44 T) orbits), estimated from the SdH oscillation in Fig. 3b, are 89 and 132 nm, respectively. Here, the $l_m$ values are estimated by $l_m = \hbar v_F/(2\pi k_BT_D)$ (where *v*_F_ is Fermi velocity, *k*_B_ is Boltzmann’s constant, and *T*_D_ is the Dingle temperature). The mean free paths of other orbits (*F*_3_–*F*_6_) are smaller than these two orbits (*F*_1_ and *F*_2_). Since the mean free paths of the Weyl fermions are larger than the thickness of the SrRuO_3_ films ($d_{{\mathrm{SrRuO}}_3}$ = 63 nm), the boundary scattering may contribute to the negative MR. However, we can exclude its contribution as described as follows. The boundary-scattering-induced MR should saturate in a high-magnetic-field region^75^, since boundary scattering does not occur when cyclotron diameter *d*_c_ (=ℏ*k*_F_/(π*eB*); *k*_F_, Fermi wave number) is smaller than the film thickness ($d_{{\mathrm{SrRuO}}_3}$ = 63 nm). If the MR had been induced by boundary scattering, it would have saturated when *B* is larger than 0.93 and 1.2 T for the *F*_1_ and *F*_2_ orbits, respectively, at which *d*_c_ becomes comparable to $d_{{\mathrm{SrRuO}}_3}$. On the contrary, the observed negative MR (Fig. 2a, c) does not show saturation behavior with *B*. Note that the coherent lengths of the other orbits (*F*_3_–*F*_6_) are not long enough to travel a full cyclotron trajectory at the measurement temperature (=2 K), where only the *F*_1_ and *F*_2_ SdH oscillation peaks are detected; as shown in Fig. 3d, the SdH oscillation peaks of *F*_3_–*F*_6_ are observed at 0.1 K. Therefore, the contribution of the boundary scattering to the negative MR (Fig. 2a, c) is negligibly small.

### Effect of the Fermi-level pinning on the phase shift in the SdH oscillation

A large number of carriers in 3D multiband systems pin the chemical potential. In 2018, Kuntsevich et al. phenomenologically explained that, even when a large number of carriers in normal bands pin the chemical potential, the SdH oscillation of the Dirac (linear) dispersion should show the π phase shift^76^. Here, we will explain why the chemical potential pinning does not affect the phase shift in the SdH oscillations, along with ref. ^76^.

We will begin with the general explanation of SdH oscillations without the Fermi-level (chemical potential) pinning. In the SdH oscillation, the minimal conductivity is obtained when the chemical potential *μ*_*c*_ is located in the mid-gap between the Landau levels (Supplementary Fig. 5a). For this *μ*_*c*_, the *N*th Landau level beneath the chemical potential is fully occupied and the density of states shows the minimal value. Reflecting this situation, in the LK theory^77^, the magnetic field at which the magnitude of the SdH oscillation takes a minimal value is expressed as

7 $$\begin{array}{*{20}{c}} {\frac{{F_i}}{{B_N^{{\mathrm{min}}}}} = N - \beta _{{\mathrm{B}}{i}}.} \end{array}$$

Here, *F*_*i*_ is the frequency of the SdH oscillation, $B_N^{\rm{min}}$ is the magnetic field giving the minimal value of the conductivity at the *N*th Landau level, and 2*πβ*_B*i*_ is the phase shift. The important point of Eq. (7) is that the $1/B_N^{{\mathrm{min}}}$ can be expressed as a linear function of *N*. This ensures the validity of using Eq. (7) for the determination of the phase shift caused by the Berry phase as we did in the main text.

Next, we consider the situation where a large number of carriers in normal bands pin the chemical potential, i.e. $\mu _c\left( B \right) = const.$ The *N*th Landau levels of the quadratic energy band ${\it{\epsilon }}_{N,Q}$ and the linear energy band ${\it{\epsilon }}_{N,D}$ are expressed as^76,78^

8 $$\begin{array}{*{20}{c}} {{\it{\epsilon }}_{N,Q} = \frac{{\hbar eB}}{m}\left( {N + \frac{1}{2}} \right)} \end{array}$$

and

9 $$\begin{array}{*{20}{c}} {{\it{\epsilon }}_{N,D} = \sqrt {2N\hbar eBv^2} ,} \end{array}$$

respectively, where *m* is an effective mass, *v* is the velocity of electrons in the linear band, and *N* is the Landau level index. The conductivity becomes maximum at $B$ with which the Landau level crosses *μ*_*c*_, since the density at *μ*_*c*_ becomes maximum when *μ*_*c*_ is located at the center of the Landau level (Supplementary Fig. 5b). Therefore, by solving the equation ${\it{\epsilon }}_{N,Q(D)} = \mu _c$, we can obtain the relationships between *N* and the magnetic field $B_{N,Q(D)}^{{\mathrm{max}}}$ at which the conductivity becomes maximum as

10 $$\begin{array}{*{20}{c}} {\hbar \frac{{eB_{N,Q}^{\rm{max}}}}{m}\left( {N + \frac{1}{2}} \right) = \mu _c = {\rm{const.}}} \end{array}$$

and

11 $$\begin{array}{*{20}{c}} {2N\hbar eB_{N,D}^{{\rm{max}}}v^2 = \mu _c^2 = {\rm{const.,}}} \end{array}$$

for the quadratic and linear bands, respectively. Since $B_{N,Q(D)}^{\rm{min}}$ is located roughly at the middle point between $B_{N - 1,Q(D)}^{{\mathrm{max}}}$ and $B_{NQ(D)}^{{\mathrm{max}}}$, we can obtain the relationship between $B_{NQ(D)}^{{\mathrm{min}}}$and *N* by shifting *N* in Eqs. (10) and (11) by a half-integer as

12 $$\begin{array}{*{20}{c}} {\frac{{\mu _cm}}{{e\hbar }}\frac{1}{{B_{N,Q}^{{\mathrm{min}}}}} = N} \end{array}$$

and

13 $$\begin{array}{*{20}{c}} {\frac{{\mu _c^2}}{{2\hbar ev^2}}\frac{1}{{B_{N,D}^{{\mathrm{min}}}}} = N - \frac{1}{2}} \end{array},$$

respectively. Then, by using the relationship between the $\mu _c$ and the Fermi wave number *k*_F_ for the quadratic and linear band, $\mu _c = \left( {\hbar k_F} \right)^2/2m$ and $\mu _c = \hbar vk_F$, respectively, and the relationship between the Fermi surface area *S* ($= \pi k_F^2$) and *F*_*i*_, $S = 2\pi eF_i/\hbar$, Eqs. (12) and (13) can be simply expressed as

14 $$\begin{array}{*{20}{c}} {\frac{{F_i}}{{B_{N,Q}^{{\mathrm{min}}}}} = N} \end{array}$$

and

15 $$\begin{array}{*{20}{c}} {\frac{{F_i}}{{B_{N,D}^{{\mathrm{min}}}}} = N - \frac{1}{2}.} \end{array}$$

Equations (14) and (15) mean that the trivial (quadratic) and nontrivial (linear) dispersion provides 0 and *π* (=2*π* × 1/2) phase shifts, respectively, even when a large number of carriers pin the chemical potential. Therefore, whether the chemical potential is pinned or not in our SrRuO_3_ film, we can estimate the phase shift by using the LK theory [Eq. (1)]. Physically, the difference in the phase shifts in Eqs. (14) and (15) comes from the difference in the Berry phases of quadratic and linear dispersions^76^.

In a 3D system with *B* applied in the *k*_*z*_ direction, a two-dimensional cyclotron motion occurs in the *k*_*x*_–*k*_*y*_ plane in 3D *k*-space at the *k*_*z*_ position where the area of the Fermi surface takes an extremal value^79^. This cyclotron orbit is called an “extremal orbit”. As in the case of 2DEG Rashba systems, graphene, and topological surface states, observation of the π Berry phase, which originates from a band touching point of the Weyl node and accumulates along the extremal orbit, is one of the important signatures of Weyl fermions^20,23,24^. Therefore, we think that the observed π Berry phase in the SdH oscillations is acquired by a surface integral of the Berry curvature *Ω* over a closed surface containing a Weyl point in *k*-space (Fig. 3a).

### Data pretreatment for quantum oscillations

Pretreatments of the SdH oscillation data are crucial for deconvoluting quantum oscillation spectra, since magnetoconductivity data generally contain not only oscillation components but also other magnetoresistive components as background signals^43^. In particular, SdH oscillations in SrRuO_3_ are subject to being masked by large non-saturated positive MR (Supplementary Fig. 5c)^43^. Here, we subtracted the background using a polynomial function up to the fifth order and extracted the oscillation components as shown in Supplementary Fig. 5d. Then, we carried out the well-established pretreatment procedure for Fourier transform of quantum oscillations^80,81^. First, we interpolated the background-subtracted data to prepare an equally spaced data set as a function of 1/*B*. Then, we multiplied the Hanning window function to obtain the periodicity of the experimental data. Finally, we performed fast Fourier transform on the treated data set.

### Exclusion of other possible mechanisms of the phase shift in SrRuO_3_

Here, we exclude other possible non-topological effects that could cause the phase shift in SdH oscillations, which are the mosaic effect, magnetic breakdown, and Zeeman splitting.

The phase shift and deviation of the SdH oscillations from the conventional LK theory, which occur at crystal grains and magnetic domains, are collectively called the mosaic effect^79,82^. This effect may occur in samples having multiple crystal domains, such as polycrystals, or in ferromagnets having magnetic domain structures. However, it should be negligible in our samples, because they are high-quality single-crystalline thin films as shown by a STEM image (Fig. 1c, Supplementary Fig. 1a, and Supplementary Fig. 5e) and they are free from magnetic domain structures as shown in Supplementary Fig. 3c where magnetization is saturated at about 0.5 T.

Next, the possibility of magnetic breakdown is also ruled out since there is no magnetic breakdown orbits in the SdH oscillations for the SrRuO_3_ film with the RRR of 84.3 (Fig. 3). Magnetic breakdown occurs when different orbits approach each other closely in *k*-space under the presence of large magnetic fields, resulting in a new orbit (a magnetic breakdown orbit) whose frequency is given by the sum of the frequencies of the original orbits^83^. If magnetic breakdown had occurred in our measurement field range (|*B*| < 14 T), we would have observed magnetic breakdown orbits whose frequencies are *F*_1_ + *F*_2_ (=70 T), *F*_1_ + *F*_3_ (=326 T), and so on. However, we did not find such frequencies in our quantum oscillation analysis as shown in Supplementary Table 1. In particular, *F*_1_ and *F*_2_, which are responsible for the non-trivial phase shifts, cannot be produced by the sum of the other orbitals’ frequencies. Therefore, magnetic breakdown is not the origin of the phase shift.

Finally, we focus on the effect of Zeeman splitting on the phase shift in a magnetic Weyl semimetal. The condition where the quantum oscillation takes minimal or maximum values is expressed as^78^

16 $$\begin{array}{*{20}{c}} {\frac{F}{H} = n + \gamma \pm \frac{1}{2}S.} \end{array}$$

Here, *F* is a frequency, *H* is a magnetic field, *n* is an integer value, *γ* indicates the phase shift caused by the Berry phase, and *S* is the spin-splitting parameter from Zeeman effect. The ± sign indicates the up/down spins in each Landau level. Due to the Zeeman effect, every Landau level is split-off by the magnetic field, and finally it affects the phase shift *γ* through the change of the *S* value. In fact, this Zeeman splitting of Landau levels changes SdH spectra in Weyl semimetals and magnetic Dirac materials^20,84^, in which Landau levels are degenerate, and this effect has to be taken into account to deduce the phase shift *γ* from the experimental data. By contrast, in SrRuO_3_, all Landau levels are not degenerate since the ferromagnetic exchange coupling lifts the spin degeneracy of all the electronic bands crossing the *E*_F_^12,42,43,85^. Altogether, since the *S* value of Eq. (16) is zero in SrRuO_3_, we can simply estimate the phase shift *γ* by the LK theory^77^, in which *S* is not taken into account, and assign the phase shift in the SdH oscillations to the Berry phase accumulation along the cyclotron orbits.

### SdH oscillations of trivial orbits

Together with SdH oscillations from the nontrivial orbits having low frequencies (*F*_1_ and *F*_2_) (Fig. 3b, c), we observed SdH oscillations from the trivial orbits having high frequencies (*F*_3_–*F*_6_) at 0.07 K < *T* < 0.75 K and 12.5 T < *B* < 14 T (Fig. 3d and Supplementary Fig. 6b). Since the carriers in the trivial orbits in SrRuO_3_ are expected to have larger effective masses than those in the *F*_1_ and *F*_2_ orbits^12,43,58^, measurements of the former oscillations should be carried out in relatively low-*T* and high-*B* regions. We estimated the cyclotron masses of the carriers in *F*_3_–*F*_6_ orbits from the temperature dependences of the respective peaks based on the LK theory (Supplementary Fig. 6c–f). In the LK theory for the mass estimation, *B* is determined as the interval value in the magnetic field range. Supplementary Table 1 shows the estimated cyclotron masses for *F*_1_–*F*_6_. The cyclotron masses in the *F*_3_–*F*_6_ orbits are relatively high (>2.8*m*_0_), reflecting the trivial band structure (energy dispersions) as their origin.

### RRR dependence of the ferromagnetism, Fermi liquid behavior, and Weyl behavior

In Fig. 4a–c, we investigated the RRR dependence of the ferromagnetism, Fermi liquid behavior, and Weyl behavior in SrRuO_3_. For the ferromagnetism, *T*_C_ values are estimated as the position of the kinks in *ρ*_*xx*_ vs. *T* curves as shown in Supplementary Fig. 7a. For the Fermi liquid behavior, we defined the Fermi liquid region (*T* < *T*_F_) as the temperature range where the experimental *ρ*_*xx*_ and the linear fitting line in *ρ*_*xx*_ vs. *T*^2^ are close enough to each other (<0.1 μΩ cm) as shown in Supplementary Fig. 7b. The upper limit temperature for measuring Weyl behavior in SrRuO_3_ (*T*_W_) is defined as the highest temperature at which the resistivity at zero field is lower than that at 9 T (*ρ*_*xx*_(0 T) < *ρ*_*xx*_(9 T)).

### RRR dependence of the Hall resistivity

Supplementary Figure 7c shows the Hall resistivity *ρ*_*xy*_(*B*) curves of the different RRR samples at 2 or 2.3 K with *B* applied in the out-of-plane [001] direction of the SrTiO_3_ substrate. As we explained in the main paper, the *ρ*_*xy*_(*B*) curves of the SrRuO_3_ film with the RRR of 84.3 at 2 K is nonlinear, indicating the coexistence of multiple types of the Weyl fermions from which the unsaturated linear positive MR stems. Notably, as shown in Supplementary Fig. 7c, *dρ*_*xy*_/*dB* changes its sign from negative to positive with decreasing RRR. In the SrRuO_3_ film with the RRR of 8.93, clear AHE is observed near zero magnetic field due to its large residual resistivity of 20.2 μΩ cm, and the *ρ*_*xy*_(*B*) curve shows the linear dependence on *B* above 5 T as highlighted in Supplementary Fig. 7c. The carrier concentration and the mobility of the holes of the SrRuO_3_ film with the RRR of 8.93, which are estimated from the slope of the *ρ*_*xy*_(*B*) above 5 T, are 4.04 × 10^22^ cm^−3^ and 7.65 cm^2^ V^−1^ s^−1^, respectively. The carrier concentration and the mobility are consistent with the reported values for the trivial Ru 4*d* bands crossing the *E*_F_ in SrRuO_3_^44,86^. These results mean that the Weyl fermions become more dominant in the transport properties with increasing RRR and that the contribution of the Weyl fermions on the Hall resistivity is negligibly small when the RRR is 8.93.

As described in the main text, the unsaturated linear positive MR also becomes more prominent with increasing RRR and decreasing temperature below *T*_F_, indicating again that the nonlinear *ρ*_*xy*_(*B*) is a hallmark of the existence of the Weyl fermions in SrRuO_3_ and that the Weyl fermions become dominant in the transport when scatterings from impurities and phonons are sufficiently suppressed.

### Computational details

Electronic structure calculations were performed within the density functional theory and generalized gradient approximation (GGA, Perdew–Burke–Ernzerhof)^87^ for the exchange correlation functional in the projector-augmented plane wave (PAW) formalism^88^ as implemented in the Vienna ab-initio Simulation package^89^. The energy cutoff was set to 500 eV, the Brillouin zone was sampled by an 8 × 8 × 6 Monkhorst–Pack mesh^90^, and the convergence criterion for the electronic density was defined as 10^−8^ eV. The effect of electronic correlations in the Ru *d* shell (4*d*^4^ for Ru^4+^) was taken into account by using the rotationally invariant GGA + *U* scheme^91^ with *U* = 2.6 eV and *J* = 0.6 eV. The choice of parameters is justified by early estimations^92^ and is in agreement with other studies^93,94^.

### First-principles calculations of Weyl points

The orthorhombic phase of SrRuO_3_ has the *Pbnm* (#62) space group, which corresponds to the *D*_*2h*_ point group with symmetries of inversion (*I*), three mirror planes (*m*_*x*_*, m*_*y*_*, m*_*z*_), and 180° rotations around the orthorhombic axes (*C*_*x*_, *C*_*y*_, *C*_*z*_). The crystal structure parameters considered in the present study are *a* = 5.5670 Å, *b* = 5.5304 Å, *c* = 7.8446 Å,^12^ and the atomic Wyckoff positions in fractional coordinates are given as 4*c* (0.5027, 0.5157, 0.25) for Sr, 4*b* (0.25, 0, 0) for Ru, and 8*d* (0.7248, 0.2764, 0.0278) for O.

The results of electronic structure calculations with and without SOC are shown in Supplementary Fig. 8. One can see that the bands close to the Fermi level are formed by the Ru 4*d* states hybridized with the O 2*p* states and the electronic spectrum reveals half-metallicity (Supplementary Fig. 8a) in agreement with previous electronic structure calculations^59^. The ferromagnetic alignment was found to be the ground state configuration with the easy axis along the orthorhombic *b* axis ($E_{010} - E_{001} =$−2.17 meV/f.u. and $E_{010} - E_{100} =$−0.35 meV/f.u.), and the calculated magnetic moments per Ru ion (Supplementary Table 2) are close to the experimental saturation magnetic moment of 1.25 $\mu _B$/Ru.^28^ The ferromagnetic state reduces the symmetry to the *C*_*2h*_ point group with one mirror plane and one rotation axis symmetry, perpendicular and parallel to the magnetization direction, respectively.

For numerical identification of the Weyl points, one needs to have a band structure with high resolution in the reciprocal space. To interpolate the resulting electronic spectrum, we employed maximally localized Wannier functions as implemented in the wannier90 package^95^. The wannierization was carried out by projecting the bands corresponding to the Ru *e*_*g*_ and *t*_*2g*_ states onto the atomic *d* orbitals in the local coordinate frame at each Ru site.

To locate the points of degeneracy between the bands in the reciprocal space, we performed a steepest-descent minimization of the gap function ${\mathrm{{\Delta}}} = (E_{n + 1,{\boldsymbol{k}}} - E_{n,{\boldsymbol{k}}})^2$on a uniform grid of up to 31 × 31 × 31 covering the Brillouin zone, where the bands are considered degenerate when the gap is below the threshold of 10^−5^ eV^96^. To eliminate accidental crossings, the corresponding chirality at each identified point is determined by evaluating the outward Berry flux enclosed in a small sphere. The calculated chiralities $\chi$ should obey the following symmetry properties: $\chi$ does not change its sign under 180° rotations around the magnetization axis and changes its sign under mirror reflection and inversion. In this study, we only consider the points with $\chi = \pm 1$.

We have selected two pairs of bands I and II for the cases when the magnetization is along the orthorhombic *c* and *b* axes, as shown in Supplementary Fig. 9 and Supplementary Fig. 10, respectively. The corresponding gap function ${\Delta} \le 0.1$ eV is calculated to demonstrate the proximity of the selected bands. From the number of the calculated band crossings, the Weyl points were identified as the ones that have close energy positions and *k*-point coordinates and whose chiralities obey the symmetry properties in the full Brillouin zone. The resulting Weyl points are listed in Supplementary Tables 3–6. Numerical differences in the coordinates of the Weyl points can be attributed to small spin canting (see Supplementary Table 2), which *slightly* breaks inversion symmetry (the reflection and rotation symmetries along to the magnetization are intact). From Supplementary Tables 3–6, most of the Weyl points are found to exist within an energy range of −0.2 to 0.2 eV around the Fermi level. In particular, $\left| {E - {E}_{\mathrm{F}}} \right|$ for WP_z_6_1–4_ (8–16 meV), which are located near the Y–T line in the Z–Γ–Y–T plane as shown in Fig. 5c, is very close to the experimental chemical potentials of the Weyl fermions estimated from the SdH oscillations (*µ*_1_ = 8.5 meV and *µ*_2_ = 8.8 meV for the *F*_1_ and *F*_2_ orbitals, respectively).

From the obtained results, one can clearly see the presence of the Weyl fermions below and above the Fermi level coexisting with trivial half-metallic bands. However, it is worth commenting on another scenario. According to previous theoretical studies^93,94^, there is no clear consensus on whether the electronic spectrum of orthorhombic SrRuO_3_ in the ferromagnetic state is half-metallic or not, and the result turns out to depend on the details of electronic structure calculations. While our theoretical results are in good agreement with the present experiments, we do not rule out the possibility of a non-half-metallic behavior with both spin channels crossing close to the Fermi level. Assuming that the spin-up states can also lie at the Fermi level, there will be an extra set of band crossings in addition to the Weyl points already defined in a half-metallic scenario.

Finally, it is worth noting that a small monoclinic distortion induced by the SrTiO_3_ substrate breaks the orthorhombic *D*_*2h*_ symmetry. The reported crystal structure parameters of epitaxial SrRuO_3_ on SrTiO_3_ are *a* = 5.5290 Å, *b* = 5.5770 Å, *c* = 7.8100 Å, $\alpha$ = 89.41°.^12^ According to our electronic structure calculations within GGA + *U* with *U* = 2.6 eV and *J* = 0.6 eV for the monoclinic SrRuO_3_ on SrTiO_3_ (Supplementary Fig. 11), the electronic spectrum does not reveal any qualitative changes from that for the orthorhombic *D*_*2h*_ symmetry, while additional band crossings may occur due to the lowered crystal symmetry.

## Supplementary information

Supplementary Information

Peer Review File

## Data Availability

The data that support the findings of this study are available from the corresponding author upon reasonable request.
