# Supplementary material for: Quantum transport evidence of Weyl fermions in an epitaxial ferromagnetic oxide
Source: Nat Commun. 2020 Oct 9;11:4969. doi: 10.1038/s41467-020-18646-8 (PMC7547107; doi:10.1038/s41467-020-18646-8)
Supplement: Supplementary file 1 — Supplementary Information [file 41467_2020_18646_MOESM1_ESM.pdf]

Supplementary Information

Quantum transport evidence of Weyl fermions in an epitaxial ferromagnetic oxide

Kosuke Takiguchi,[1,2,†] Yuki K. Wakabayashi,[1,*,†] Hiroshi Irie,[1] Yoshiharu Krockenberger,[1] Takuma Otsuka,[3] Hiroshi Sawada,[3] Sergey A. Nikolaev,[4,5] Hena Das,[4,5] Masaaki Tanaka,[2] Yoshitaka Taniyasu,[1] and Hideki Yamamoto[1]

[1]*NTT Basic Research Laboratories, NTT Corporation, Atsugi, Kanagawa 243-0198, Japan.*

[2]*Department of Electrical Engineering and Information Systems & Center for Spintronics Research Network (CSRN), The University of Tokyo, 7-3-1 Hongo, Bunkyo-ku, Tokyo 113-8656, Japan.*

[3]*NTT Communication Science Laboratories, NTT Corporation, Soraku-gun, Kyoto 619-0237, Japan.*

[4]*Laboratory for Materials and Structures, Tokyo Institute of Technology, 4259 Nagatsuta, Midori-ku, Yokohama, Kanagawa 226-8503, Japan.*

[5]*Tokyo Tech World Research Hub Initiative (WRHI), Institute of Innovative Research, Tokyo Institute of Technology, 4259 Nagatsuta, Midori-ku, Yokohama, Kanagawa 226-8503, Japan.*

*email: yuuki.wakabayashi.we@hco.ntt.co.jp

†These authors contributed equally to this work.

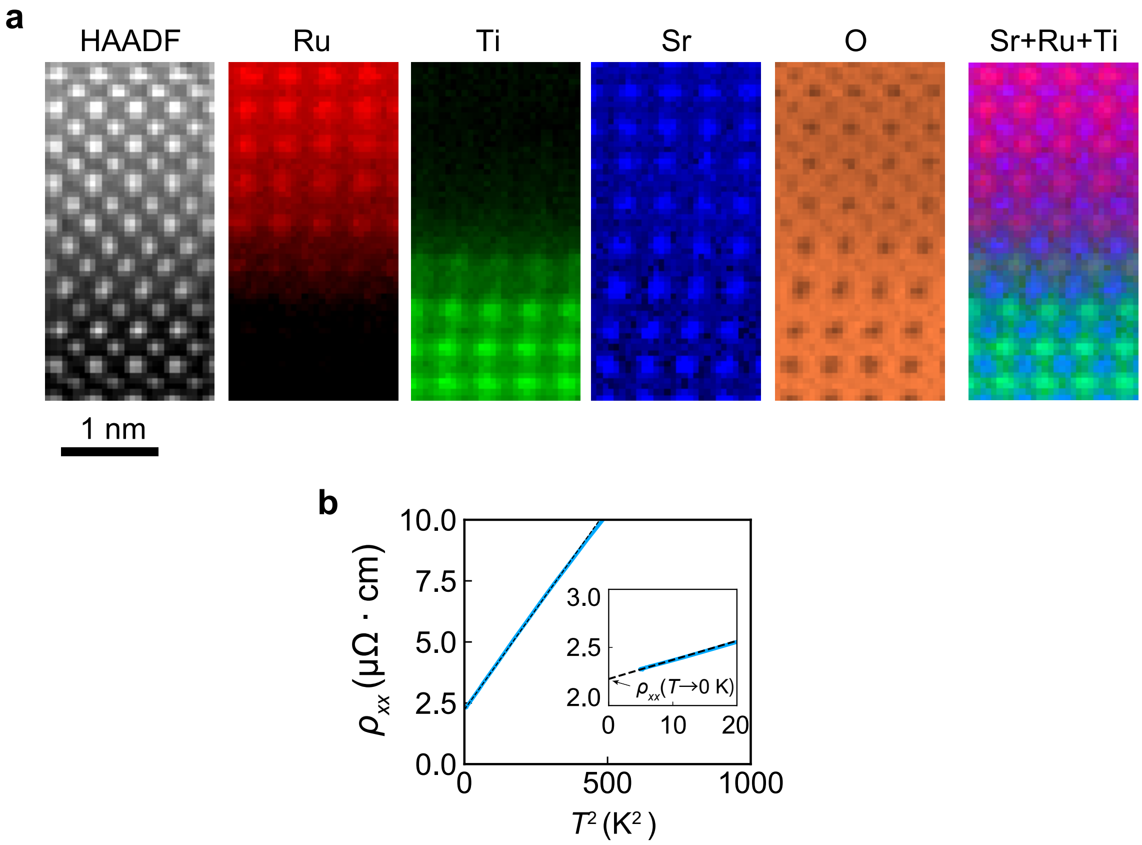

Supplementary Fig. 1 HAADF-STEM and EELS-STEM images, and Fermi liquid behaviour in $SrRuO_3$. a, (From left to right) HAADF-STEM image of the $SrRuO_3$ film with the RRR of 71 taken along the [100] axis of the $SrTiO_3$ substrate. EELS-STEM images for the Ru-$M_{4,5}$- (red), Ti-$L_{2,3}$- (green), Sr-M_3- (blue), O-K-edge (orange), and a color overlay of the EELS-STEM images for Sr, Ru and Ti. **b,** $\rho_{xx}(T)$ versus T^2 curve (blue line) for the $SrRuO_3$ film with the RRR of 84.3. The black dashed line is the linear fitting result. Close-up near 0 K^2 is shown in the inset. The $\rho_{xx}(T\rightarrow 0\ K)$ value is estimated from the extrapolation of the fitting line to the 0 K^2 axis.

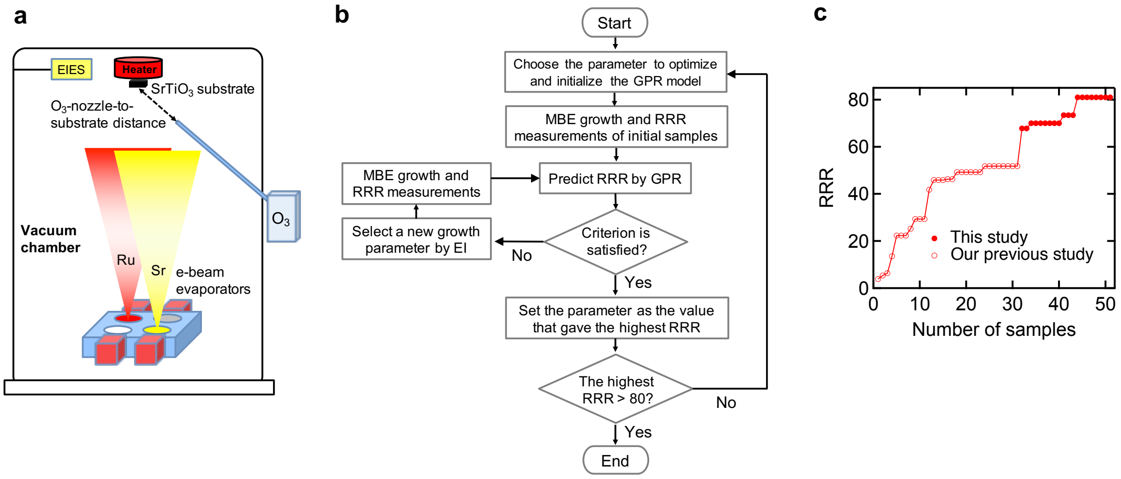

Supplementary Fig. 2 Machine-learning-assisted MBE. **a**, Schematic illustration of our multi-source oxide MBE system. EIES: electron impact emission spectroscopy. **b**, Flowchart of machine-learning-assisted MBE growth based on the BO algorithm. **c**, Highest experimental RRR values plotted as a function of the total number of MBE growth runs. In **c**, open circles are data deduced from Ref. 28. Here, the Ru flux rate, growth temperature, and nozzle-to-substrate distance were varied in ranges between 0.18 and 0.61 Ås^{-1}, 565 and 815°C, and 1 and 31 mm, in correspondence to the search ranges in BO.

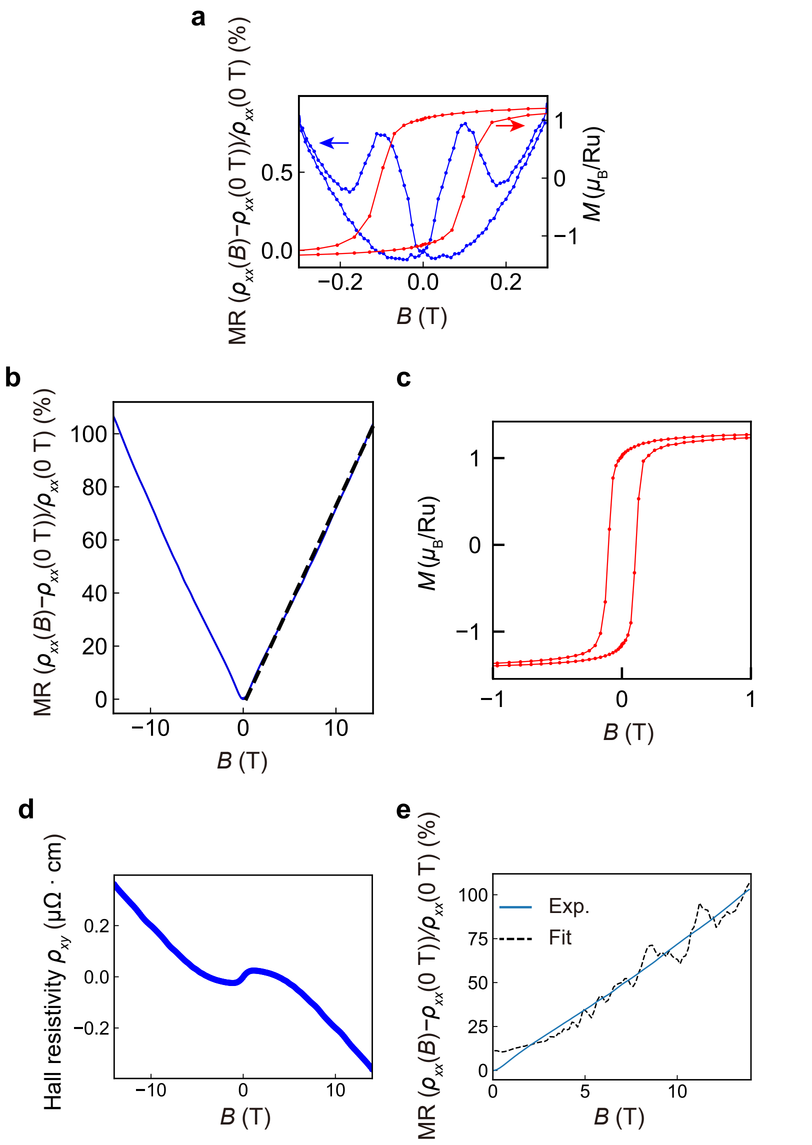

Supplementary Fig. 3 Anisotropic MR and linear positive MR in $SrRuO_3$ a, MR ($\rho_{xx}(B)$−ρ_{xx}(0 T))/ρ_{xx}(0 T) at 2 K for the $SrRuO_3$ film with the RRR of 84.3 with -0.3 T < B < 0.3 T applied in the out-of-plane [001] direction of the $SrTiO_3$ substrate (blue filled circles). Magnetization M versus B (-0.3 T < B < 0.3 T) curve at 10 K with B applied in the out-of-plane [001] direction of the $SrTiO_3$ substrate (red filled circles). **b**, MR ($\rho_{xx}(B)$−ρ_{xx}(0 T))/ρ_{xx}(0 T) at 2 K for the $SrRuO_3$ film with the RRR of 84.3 with −14 T < B < 14 T applied in the out-of-plane [001] direction of the $SrTiO_3$ substrate. The black dashed line is an eye-guide to indicate the linearity of the MR. These are the same data in Fig. 1e in the main manuscript. **c,** Magnetization M versus B (-1 T < B < 1 T) curve at 10 K with B applied in the out-of-plane [001] direction of the $SrTiO_3$ substrate. **d,** Hall resistivity $\rho_{xy}(B)$ curve at 2 K for the $SrRuO_3$ film with the RRR of 84.3 with B applied in the out-of-plane [001] direction of the $SrTiO_3$ substrate. **e,** MR ($\rho_{xx}(B)$ − ρ_{xx}(0 T))/ρ_{xx}(0 T) observed from 0 to 14 T at 2 K (light blue line) and the fitting result by eq. (2) (black dashed line). In **d** and **e**, $\rho_{xy}(B)$ and $\rho_{xx}(B)$ are the same data in Fig. 1f and 1e, respectively. The oscillating behaviour of the fitting curve is due to the quantum oscillation of $\rho_{xy}(B)$.

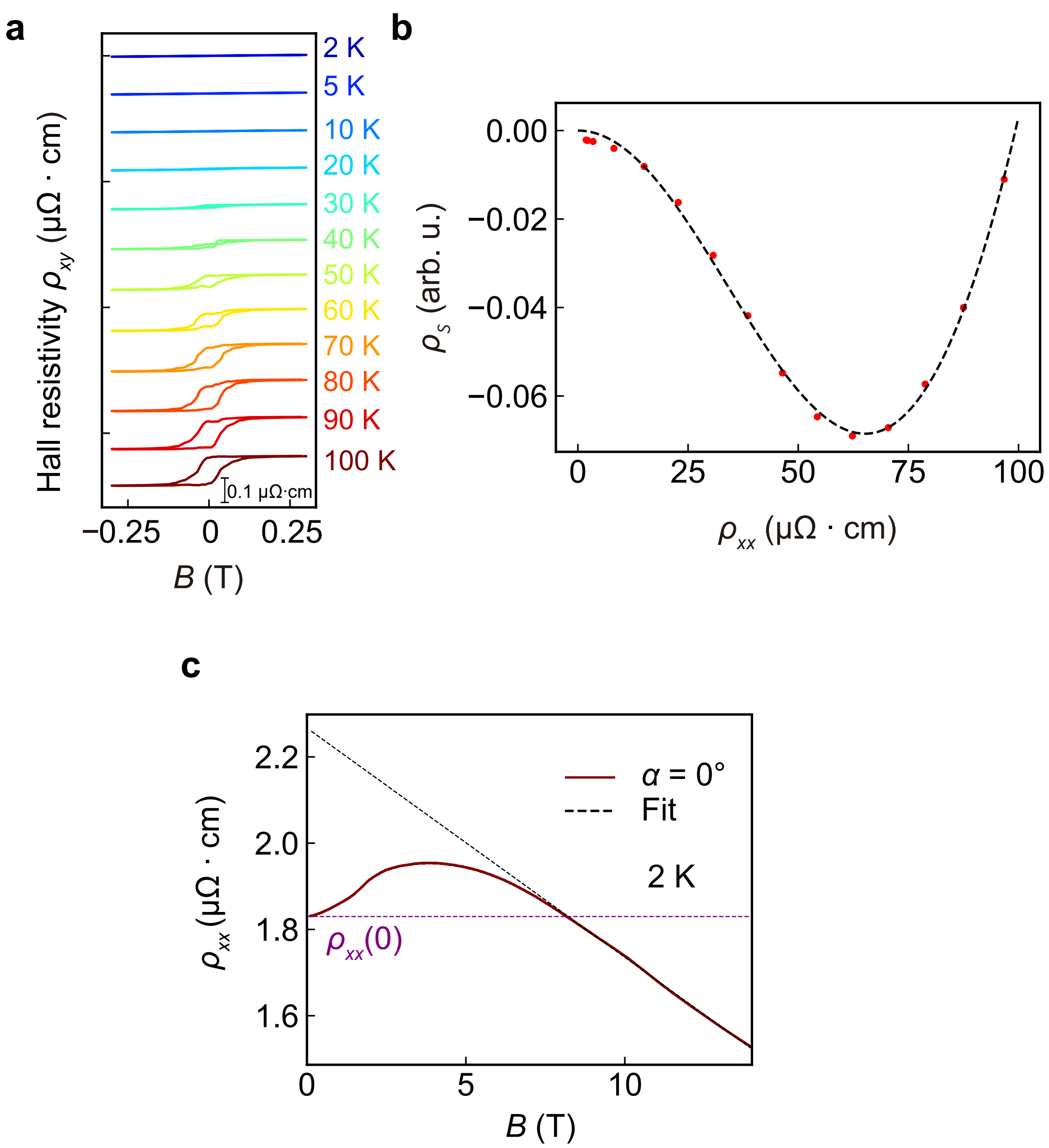

Supplementary Fig. 4 Temperature dependence of the Hall resistivity and chiral-anomaly-induced linear and negative MR. a, Hall resistivity $\rho_{xy}(B)$ curves at 2 to 100 K for the $SrRuO_3$ film with the RRR of 84.3 with -0.3 T < B < 0.3 T applied in the out-of-plane [001] direction of the $SrTiO_3$ substrate. In **a**, the Hall resistivity at each temperature has been offset by 0.15 μΩ·cm for easy viewing. **b,** ρ_s versus ρ_{xx} plot (red circles) and fitting results by eq. (4) (black dashed curve) up to 130 K. In **b**, ρ_s at each temperature was obtained by dividing ρ_{xy}(0 T) in Fig. 1f by $M_{\perp}$ with 100 Oe obtained by the SQUID measurements. Here, the magnitude of ρ_{xy}(0 T) is defined as the averaged absolute value of ρ_{xy} at ± 0.2 T. ρ_{xx} at each temperature is the same data as in Fig. 1d. **c,** $\rho_{xx}(B)$ at $\alpha = 0$ (brown solid curve) for the $SrRuO_3$ film with the RRR of 84.3 and the linear fitting line (black dashed line) to $\rho_{xx}(B)$ in the negative MR region (8 T < B < 14 T). The fitting line completely reproduces the negative and linear MR region. The purple dashed line corresponds to the value of the zero field resistivity ρ_{xx}(0 T).

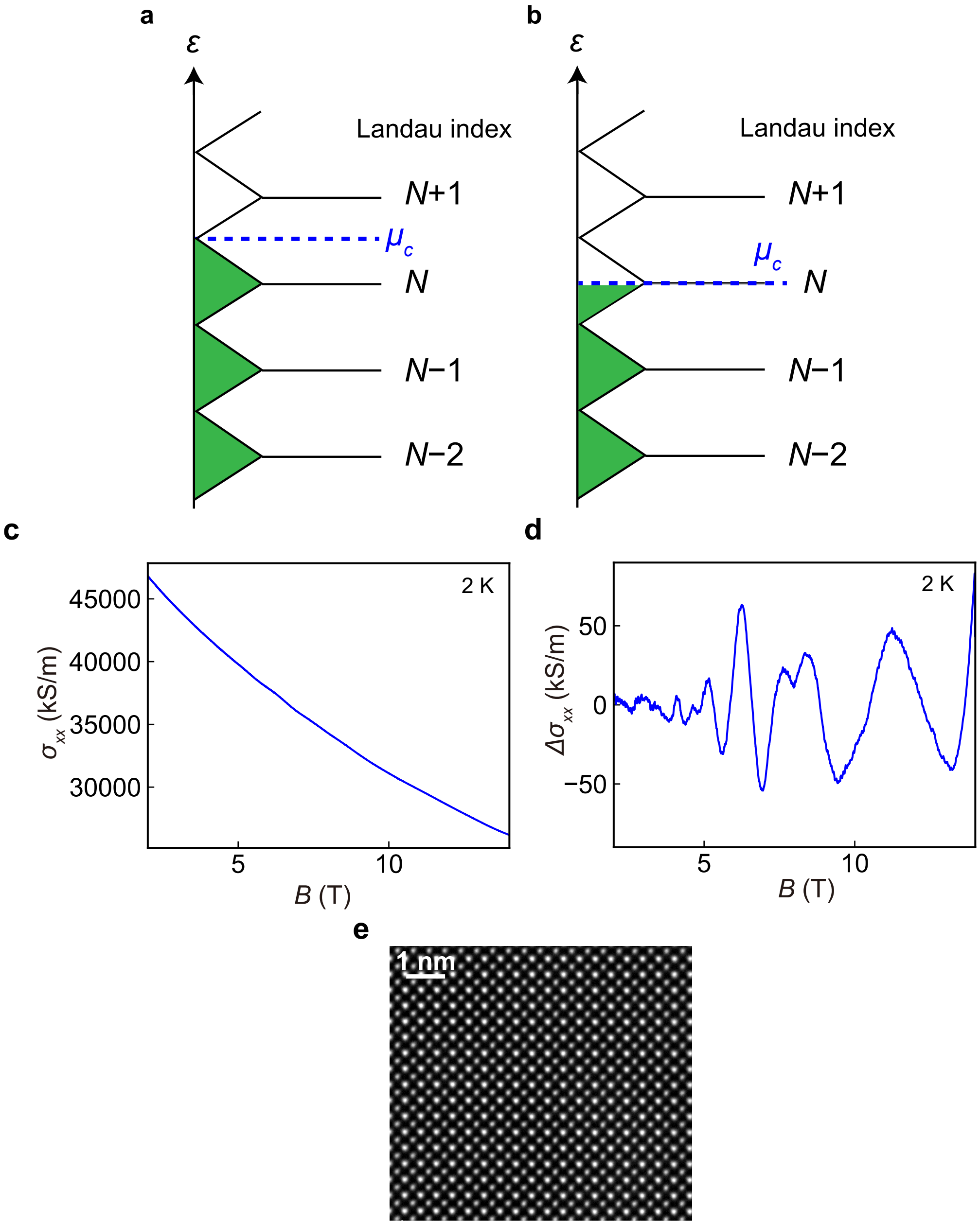

Supplementary Fig. 5 Landau quantization and chemical potential, pretreatment of the SdH oscillation data and STEM image. a, **b,** Schematic energy diagram of the relationship between Landau quantized levels ϵ_N and chemical potential μ_c which is located at the middle point between the Nth and (N+1)th Landau level, and at the center of Nth Landau level, respectively. The green region represents the filling ratio of each Landau level. In **a** and **b**, the Nth Landau level is fully and half occupied, respectively. **c,** Raw $\sigma_{xx}(B)$ data measured at 2 K and $\beta = \gamma = 90°$ for the $SrRuO_3$ film with the RRR of 84.3. The raw conductivity data is obtained by using $\rho_{xx}(B)$ and $\rho_{xy}(B)$ data as $\sigma_{xx}(B) = \rho_{xx}(B)/((\rho_{xx}(B))^2 + (\rho_{xy}(B))^2)$. **d,** SdH oscillation data $\Delta\sigma_{xx}(B)$ obtained by subtracting a polynomial function up to the fifth order from $\sigma_{xx}(B)$ in **c**. **e,** Cross-sectional high-angle annular dark field scanning transmission electron microscopy (HAADF-STEM) image of the $SrRuO_3$ film with the RRR of 71 taken along the [100] axis of the $SrTiO_3$ substrate.

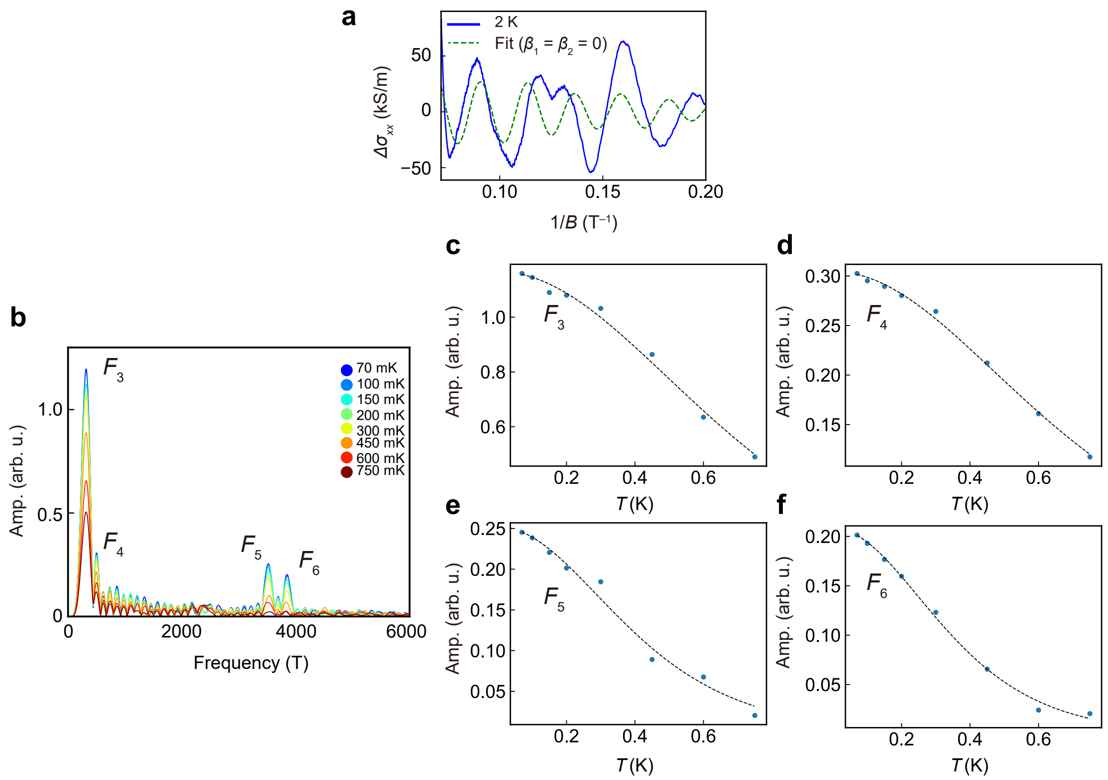

Supplementary Fig. 6 LK theory fitting to the SdH oscillations with the fixed zero Berry phase and mass estimations of the trivial orbits. a, SdH oscillation data at 2 K for the $SrRuO_3$ film with the RRR of 84.3 and the fitting results by eq. (1) with the zero Berry phases ($\beta_1 = \beta_2 = 0$). The SdH oscillation is the same data as in Fig. 3b. The fitting was carried out by a non-linear least squares method with the fitting parameters A_1, T_{D1}, A_2, and T_{D2}. The fitting curve cannot reproduce the experimental data well, confirming the existence of the non-zero Berry phase. **b,** Fourier transform spectra of the SdH oscillations from 70 to 750 mK for the $SrRuO_3$ film with the RRR of 84.3. The spectra are obtained by fast Fourier transform for the oscillation data $\Delta\rho_{xx}(B)$ from 12.5 T to 14 T. F_3, F_4, F_5, and F_6 peaks correspond to 300, 500, 3500, and 3850 T, respectively. **c-f,** Mass estimations for the F_3, F_4, F_5, and F_6 orbits according to the LK theory. Black dashed curves are fitting curves.

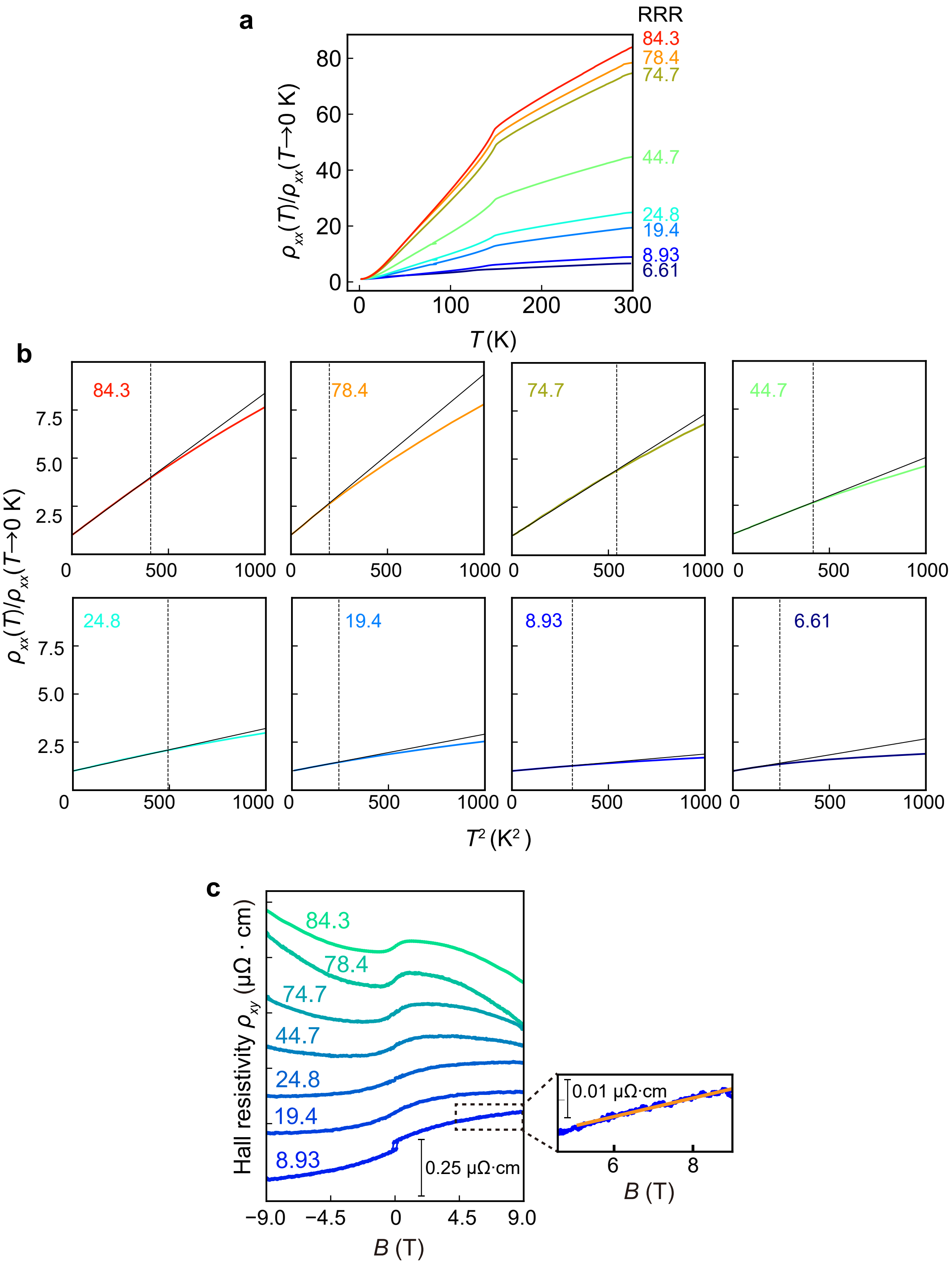

Supplementary Fig. 7 RRR dependence of T_C, T_F, and Hall resistivity. a, $\rho_{xx}(T)/\rho_{xx}(T\to0$ K) versus T of the different RRR samples. The kinks around 150 K correspond to T_C of the samples. **b,** $\rho_{xx}(T)/\rho_{xx}$ ($T\to0$ K) versus T^2 curves with the linear fittings (black lines) for the different RRR samples. The black dashed lines correspond to T_F^2 where the experimental ρ_{xx} and the fitting line are close enough to each other (< 0.1 μΩ cm). **c,** Hall resistivity $\rho_{xy}(B)$ curves of the different RRR samples at 2 or 2.3 K with B applied in the out-of-plane [001] direction of the $SrTiO_3$ substrate. The right-side figure is enlarged graph of $\rho_{xy}(B)$ with RRR = 8.93 at 4.5 T < B < 9 T. The orange line is the linear fitting result from 5T to 9 T for estimating the carrier density and mobility.

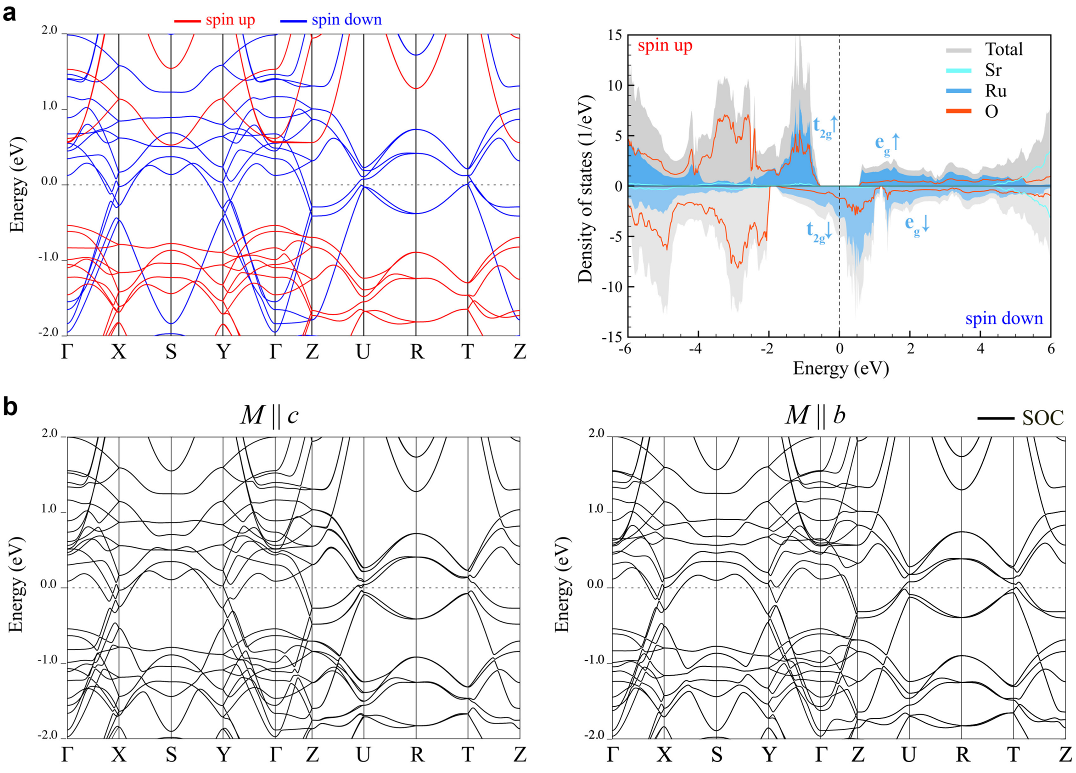

Supplementary Fig. 8 Electronic structure of orthorhombic $SrRuO_3$ calculated within GGA+*U* with *U* = 2.6 eV and *J* = 0.6 eV. a, Band structure (left) and density of states (right) for the ferromagnetic state without SOC. **b**, Band structure for the ferromagnetic state with SOC and the magnetization along the orthorhombic *c* and *b* axes (left and right, respectively) calculated within GGA+*U*+SOC. Fractional coordinates of the high-symmetry *k*-points are Γ (0,0,0), X (0.5,0,0), S (0.5,0.5,0), Y (0,0.5,0), Z (0,0,0.5), U (0.5,0,0.5), R (0.5,0.5,0.5), and T (0,0.5,0.5).

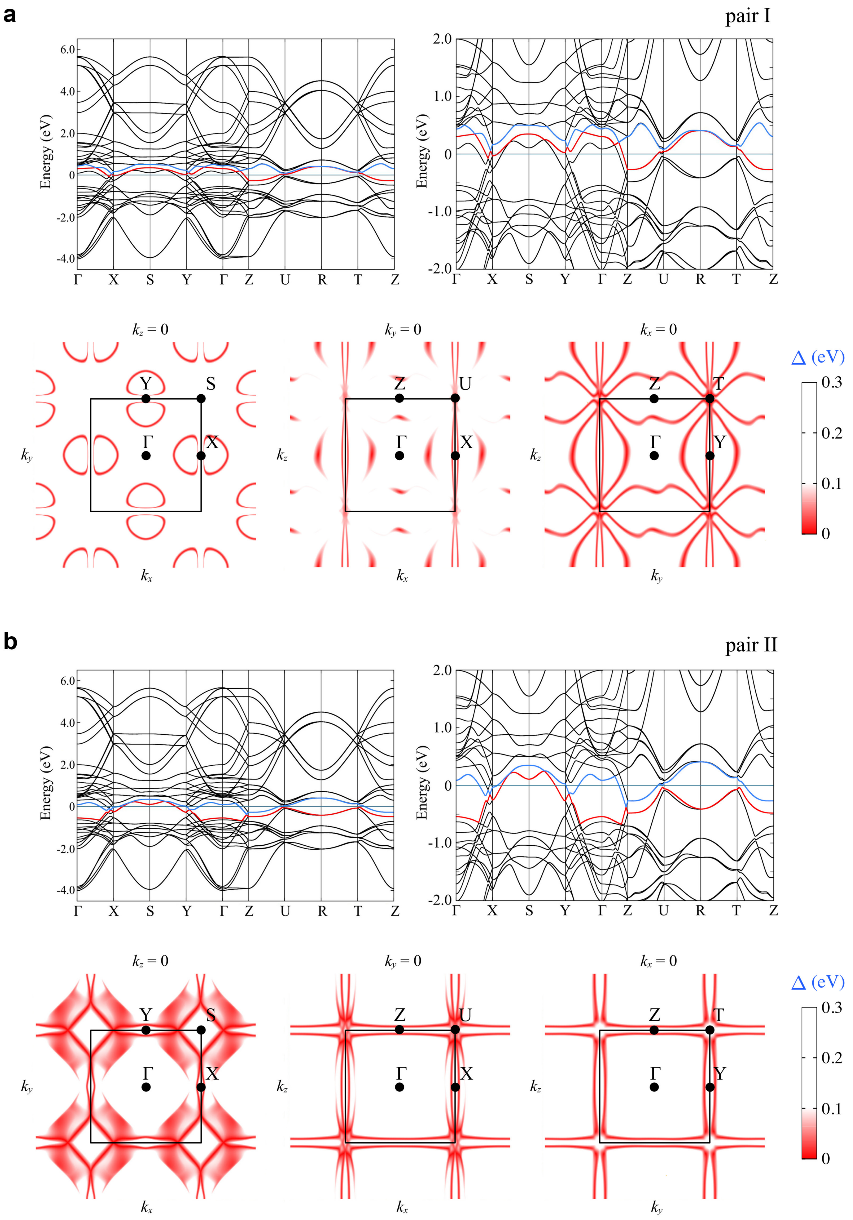

Supplementary Fig. 9 Pairs of bands I and II in the case of magnetization along the orthorhombic *c* axis. a, Gap function $\Delta \leq 0.1$ eV for the pair I (red and blue bands in upper figures in **a**) in the $k_z = 0$, $k_y = 0$, $k_x = 0$ planes. **b**, Gap function $\Delta \leq 0.1$ eV for the pair II (red and blue bands in upper figures in **b**) in the k_z=0, k_y=0, k_x=0 planes. The band structure is interpolated in the Wannier basis representing the t_{2g} and e_g states based on the electronic structure calculated within GGA+*U*+SOC.

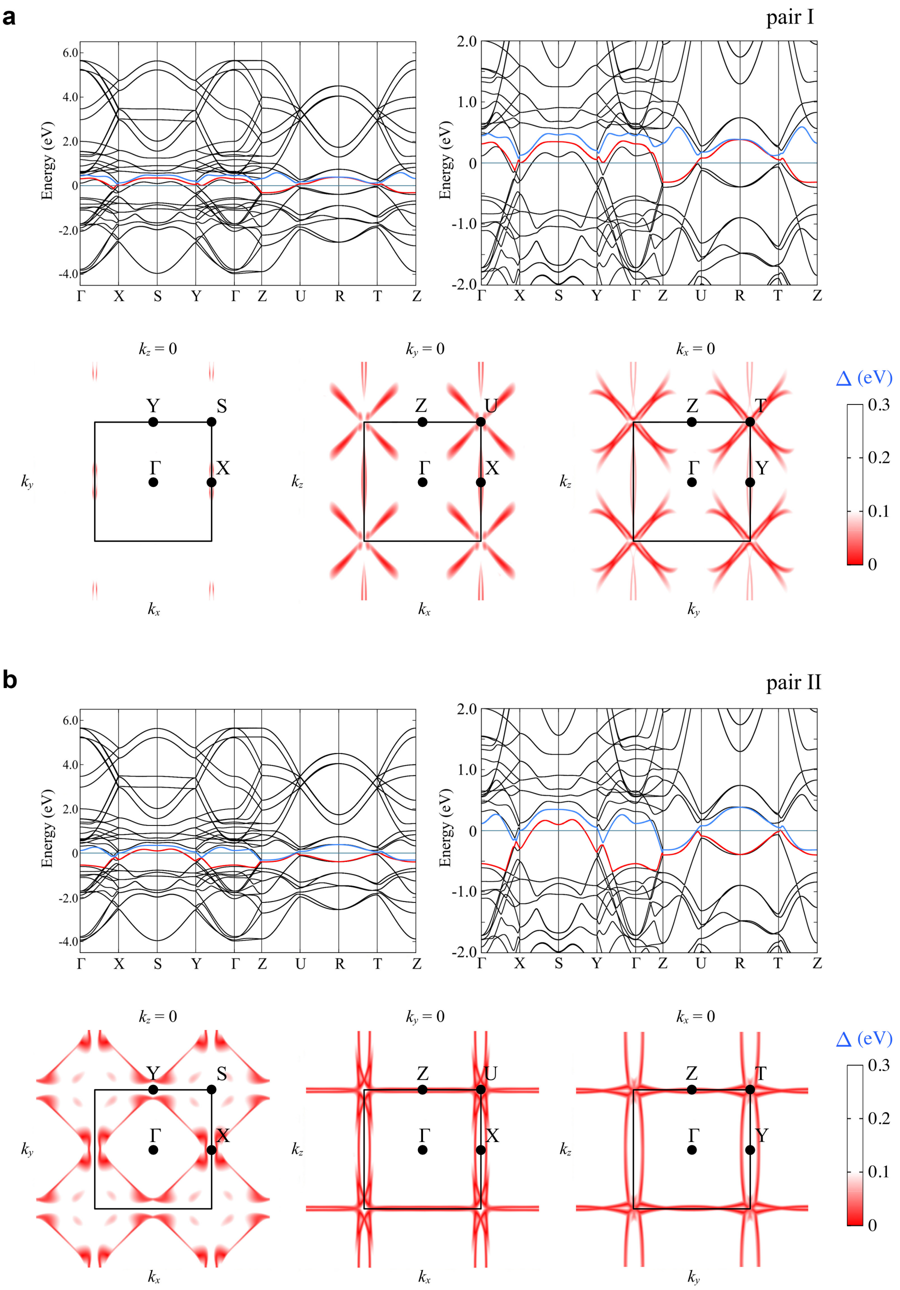

Supplementary Fig. 10 Pairs of bands I and II in the case of magnetization along the orthorhombic *b* axis. a, Gap function $\Delta \leq 0.1$ eV for the pair I (red and blue bands in upper figures in **a**) in the $k_z = 0$, $k_y = 0$, $k_x = 0$ planes. **b**, Gap function $\Delta \leq 0.1$ eV for the pair II (red and blue bands in upper graphs in **b**) in the $k_z = 0$, $k_y = 0$, $k_x = 0$ planes. The band structure is interpolated in the Wannier basis representing the t_{2g} and e_g states based on the electronic structure calculated within GGA+U+SOC.

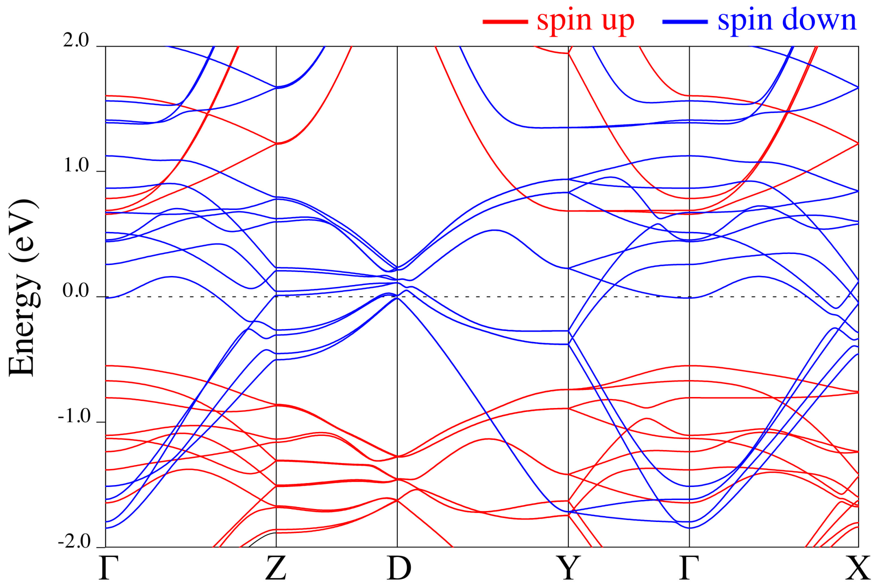

Supplementary Fig. 11 Electronic structure of monoclinic $SrRuO_3$ in the ferromagnetic state without SOC calculated within GGA+*U* with *U* = 2.6 eV and *J* = 0.6 eV. Fractional coordinates of the high-symmetry *k*-points are Γ (0,0,0), Z (0.5,0,0), D (0.5,0,0.5), Y (0,0,0.5), and X (0,0.5,0).

Supplementary Table 1 Frequencies and effective cyclotron masses estimated from SdH oscillations in the $SrRuO_3$ film with RRR = 84.3. Frequencies and effective cyclotron masses estimated in Fig. 3c and Supplementary Fig. 6c-f. m_0 represents the free electron mass in a vacuum.

	F_1	F_2	F_3	F_4	F_5	F_6
F (T)	26	44	300	500	3500	3850
m^*/m_0	0.35	0.58	2.9	3.1	5.0	5.8

Supplementary Table 2 Magnetic moments (in μ_B) of $SrRuO_3$ in the ferromagnetic state as obtained from GGA+*U*+SOC. Second and third columns show the calculated magnetic moments in μ_B units when the magnetization is along the orthorhombic c and b axes, respectively. ***S*** and ***L*** are spin and orbital magnetic moments, respectively. Ru sites are given in fractional coordinates.

Ru site	$M \parallel c$	$M \parallel b$
(0.5, 0, 0)	***S*** = (0.064, 0.010, 1.399)<br>***L*** = (0, -0.005, 0.074)	***S*** = (0.057, 1.398, -0.020)<br>***L*** = (0.003, 0.099, -0.008)
(0, 0.5, 0)	***S*** = (-0.064, 0.010, 1.400)<br>***L*** = (0, -0.005, 0.074)	***S*** = (-0.056, 1.400, -0.021)<br>***L*** = (-0.003, 0.099, -0.008)
(0.5, 0, 0.5)	***S*** = (-0.064, -0.010, 1.397)<br>***L*** = (0, 0.005, 0.074)	***S*** = (0.059, 1.397, -0.020)<br>***L*** = (0.003, 0.099, 0.008)
(0.5, 0, 0.5)	***S*** = (0.064, -0.010, 1.396)<br>***L*** = (0, 0.005, 0.074)	***S*** = (-0.057, 1.399, -0.021)<br>***L*** = (-0.003, 0.099, 0.008)

Supplementary Table 3 Weyl points calculated for the pair of bands I for the case of the magnetization along the *c* axis. The *k*-points are given in fractional coordinates.

#	E-E_F, (eV)	(k_x, k_y, k_z)	Chirality
WP_z1_1	0.204	(0, 0, 0.332)	1
WP_z1_2	0.204	(0, 0, -0.332)	-1
WP_z2_1	0.214	(0, 0.307, 0.357)	-1
WP_z2_2	0.219	(0, 0.305, -0.355)	1
WP_z2_3	0.219	(0, -0.305, 0.355)	-1
WP_z2_4	0.214	(0, -0.307, -0.357)	1
WP_z3_1	0.117	(0, 0.485, 0.456)	-1
WP_z3_2	0.116	(0, -0.484, 0.456)	-1
WP_z3_3	0.116	(0, 0.484, -0.456)	1
WP_z3_4	0.117	(0, -0.485, -0.456)	1
WP_z4_1	0.102	(-0.022, 0.439, -0.478)	-1
WP_z4_2	0.102	(0.020, 0.439, -0.478)	-1
WP_z4_3	0.101	(-0.027, 0.437, 0.479)	1
WP_z4_4	0.101	(0.029, 0.436, 0.479)	1
WP_z4_5	0.102	(-0.021, -0.439, 0.479)	1
WP_z4_6	0.102	(0.023, -0.438, 0.479)	1
WP_z4_7	0.101	(0.027, -0.437, -0.479)	-1
WP_z4_8	0.101	(-0.029, -0.436, -0.479)	-1
WP_z5_1	0.089	(0.235, -0.306, 0.483)	-1
WP_z5_2	0.087	(0.233, 0.306, 0.483)	-1
WP_z5_3	0.087	(0.233, -0.306, -0.483)	1
WP_z5_4	0.089	(0.235, 0.306, -0.483)	1
WP_z5_5	0.087	(-0.233, 0.306, 0.483)	-1
WP_z5_6	0.087	(-0.233, -0.306, -0.483)	1
WP_z5_7	0.089	(-0.235, 0.306, -0.483)	1
WP_z5_8	0.089	(-0.235, -0.306, 0.483)	-1

Supplementary Table 4 Weyl points calculated for the pair of bands II for the case of the magnetization along the *c* axis. The *k*-points are given in fractional coordinates.

#	E-E_F, (eV)	(k_x, k_y, k_z)	Chirality
WP_z6_1	-0.008	(0, 0.464, 0.400)	1
WP_z6_2	-0.016	(0, 0.464, -0.400)	-1
WP_z6_3	-0.016	(0, -0.464, 0.400)	1
WP_z6_4	-0.008	(0, -0.464, -0.400)	-1
WP_z7_1	-0.104	(0.452, 0, 0.385)	-1
WP_z7_2	-0.104	(-0.452, 0, -0.385)	1
WP_z7_3	-0.122	(0.450, 0, -0.368)	1
WP_z7_4	-0.122	(-0.450, 0, 0.368)	-1
WP_z8_1	0.239	(0.276, 0.485, -0.205)	1
WP_z8_2	0.225	(0.266, 0.483, 0.223)	-1
WP_z8_3	0.220	(0.262, -0.483, 0.228)	-1
WP_z8_4	0.231	(0.270, -0.484, -0.215)	1
WP_z8_5	0.238	(-0.275, -0.485, 0.209)	-1
WP_z8_6	0.230	(-0.269, 0.484, 0.218)	-1
WP_z8_7	0.220	(-0.262, 0.483, -0.228)	1
WP_z8_8	0.227	(-0.267, -0.484, -0.220)	1
WP_z9_1	0.142	(-0.492, 0.187, 0.170)	-1
WP_z9_2	0.142	(0.492, 0.188, 0.170)	-1
WP_z9_3	0.151	(0.492, -0.191, 0.175)	-1
WP_z9_4	0.149	(-0.492, -0.191, 0.174)	-1
WP_z9_5	0.142	(-0.491, 0.193, -0.176)	1
WP_z9_6	0.142	(-0.492, -0.188, -0.171)	1
WP_z9_7	0.149	(0.492, -0.188, -0.171)	1
WP_z9_8	0.150	(0.492, 0.192, -0.175)	1
WP_z10_1	0.257	(0.408, -0.379, -0.121)	-1
WP_z10_2	0.257	(0.426, 0.362, -0.123)	-1
WP_z10_3	0.257	(0.425, -0.363, 0.124)	1
WP_z10_4	0.258	(-0.412, 0.374, 0.128)	1
WP_z10_5	0.258	(-0.412, -0.375, -0.129)	-1
WP_z10_6	0.256	(-0.427, 0.360, -0.116)	-1
WP_z10_7	0.258	(0.403, 0.384, 0.119)	1
WP_z10_8	0.256	(-0.428, -0.359, 0.116)	1

Supplementary Table 5 Weyl points calculated for the pair of bands I for the case of the magnetization along the *b* axis. The *k*-points are given in fractional coordinates.

#	E-E_F, (eV)	(k_x, k_y, k_z)	Chirality
WP_y1_1	0.160	(0, 0.378, 0.395)	1
WP_y1_2	0.152	(0, 0.379, -0.394)	1
WP_y1_3	0.160	(0, -0.378, -0.395)	-1
WP_y1_4	0.152	(0, -0.379, 0.394)	-1
WP_y2_1	0.126	(0.485, -0.095, 0)	1
WP_y2_2	0.128	(-0.486, -0.097, 0)	1
WP_y2_3	0.128	(0.486, 0.097, 0)	-1
WP_y2_4	0.152	(-0.485, 0.095, 0)	-1
WP_y3_1	0.275	(0, -0.326, -0.297)	-1
WP_y3_2	0.275	(0, 0.326, 0.297)	1
WP_y3_3	0.282	(0, 0.321, -0.294)	1
WP_y3_4	0.281	(0, -0.321, 0.294)	-1
WP_y4_1	0.168	(0.323, -0.081, -0.375)	-1
WP_y4_2	0.163	(0.331, 0.076, -0.379)	1
WP_y4_3	0.165	(0.329, -0.077, 0.377)	-1
WP_y4_4	0.168	(-0.323, 0.082, 0.375)	1
WP_y4_5	0.165	(-0.329, 0.077, -0.377)	1
WP_y4_6	0.163	(-0.331, -0.076, 0.379)	-1
WP_y4_7	0.177	(0.306, 0.092, 0.368)	1
WP_y4_8	0.177	(-0.306, -0.092, -0.368)	-1
WP_y5_1	0.113	(-0.172, 0.308, -0.437)	-1
WP_y5_2	0.112	(0.174, -0.306, 0.437)	1
WP_y5_3	0.098	(0.196, -0.307, -0.452)	1
WP_y5_4	0.103	(0.191, 0.305, 0.449)	-1
WP_y5_5	0.099	(-0.196, 0.303, 0.451)	-1
WP_y5_6	0.102	(-0.193, -0.304, -0.450)	1
WP_y5_7	0.096	(-0.201, -0.302, 0.454)	1
WP_y5_8	0.096	(0.201, 0.302, -0.454)	-1

Supplementary Table 6 Weyl points calculated for the pair of bands II for the case of the magnetization along the *b* axis. The *k*-points are given in fractional coordinates.

#	E-E_F, (eV)	(k_x, k_y, k_z)	Chirality
$WP_{y}6_1$	-0.070	(0, 0.441, -0.349)	1
$WP_{y}6_2$	-0.060	(0, 0.441, 0.350)	1
$WP_{y}6_3$	-0.070	(0, -0.441, 0.349)	-1
$WP_{y}6_4$	-0.060	(0, -0.441, -0.349)	-1
$WP_{y}7_1$	-0.318	(0.155, -0.328, 0)	1
$WP_{y}7_2$	-0.331	(0.169, 0.313, 0)	-1
$WP_{y}7_3$	-0.320	(-0.157, 0.326, 0)	-1
$WP_{y}7_4$	-0.331	(-0.168, -0.314, 0)	1
$WP_{y}8_1$	-0.243	(-0.398, -0.101, 0)	-1
$WP_{y}8_2$	-0.260	(-0.391, 0.104, 0)	1
$WP_{y}8_3$	-0.240	(0.399, 0.100, 0)	1
$WP_{y}8_4$	-0.260	(0.390, -0.104, 0)	-1
$WP_{y}9_1$	-0.160	(0.454, 0.020, 0)	-1
$WP_{y}9_2$	-0.160	(-0.454, -0.021, 0)	1
$WP_{y}9_3$	-0.153	(-0.455, 0.026, 0)	-1
$WP_{y}9_4$	-0.154	(0.455, -0.025, 0)	1
