# Supplementary material for: Quantum transport evidence of Weyl fermions in an epitaxial ferromagnetic oxide
Source: Nat Commun. 2020 Oct 9;11:4969. doi: 10.1038/s41467-020-18646-8 (PMC7547107; doi:10.1038/s41467-020-18646-8)
Supplement: Supplementary file 2 — Peer Review File [file 41467_2020_18646_MOESM2_ESM.pdf]

Editorial Note: This manuscript has been previously reviewed at another journal that is not operating a transparent peer review scheme. The manuscript was considered suitable for publication without further review at Nature Communications .
